# Parameter estimation with bio-inspired meta-heuristic optimization: modeling the dynamics of endocytosis

**DOI:** 10.1186/1752-0509-5-159

**Published:** 2011-10-11

**Authors:** Katerina Tashkova, Peter Korošec, Jurij Šilc, Ljupčo Todorovski, Sašo Džeroski

**Affiliations:** 1 Computer Systems Department, Jožef Stefan Institute, Jamova cesta 39, SI-1000 Ljubljana, Slovenia; 2 Faculty of Administration, University of Ljubljana, Gosarjeva ulica 5, SI-1000 Ljubljana, Slovenia; 3 Department of Knowledge Technologies, Jožef Stefan Institute, Jamova cesta 39, SI-1000 Ljubljana, Slovenia

## Abstract

**Background:**

We address the task of parameter estimation in models of the dynamics of biological systems based on ordinary differential equations (ODEs) from measured data, where the models are typically non-linear and have many parameters, the measurements are imperfect due to noise, and the studied system can often be only partially observed. A representative task is to estimate the parameters in a model of the dynamics of endocytosis, i.e., endosome maturation, reflected in a cut-out switch transition between the Rab5 and Rab7 domain protein concentrations, from experimental measurements of these concentrations. The general parameter estimation task and the specific instance considered here are challenging optimization problems, calling for the use of advanced meta-heuristic optimization methods, such as evolutionary or swarm-based methods.

**Results:**

We apply three global-search meta-heuristic algorithms for numerical optimization, i.e., differential ant-stigmergy algorithm (DASA), particle-swarm optimization (PSO), and differential evolution (DE), as well as a local-search derivative-based algorithm 717 (A717) to the task of estimating parameters in ODEs. We evaluate their performance on the considered representative task along a number of metrics, including the quality of reconstructing the system output and the complete dynamics, as well as the speed of convergence, both on real-experimental data and on artificial pseudo-experimental data with varying amounts of noise. We compare the four optimization methods under a range of observation scenarios, where data of different completeness and accuracy of interpretation are given as input.

**Conclusions:**

Overall, the global meta-heuristic methods (DASA, PSO, and DE) clearly and significantly outperform the local derivative-based method (A717). Among the three meta-heuristics, differential evolution (DE) performs best in terms of the objective function, i.e., reconstructing the output, and in terms of convergence. These results hold for both real and artificial data, for all observability scenarios considered, and for all amounts of noise added to the artificial data. In sum, the meta-heuristic methods considered are suitable for estimating the parameters in the ODE model of the dynamics of endocytosis under a range of conditions: With the model and conditions being representative of parameter estimation tasks in ODE models of biochemical systems, our results clearly highlight the promise of bio-inspired meta-heuristic methods for parameter estimation in dynamic system models within system biology.

## Background

Reconstructing the structure and behavior of biological systems is of fundamental importance to the field of system biology. In general, biological systems exhibit complex nonlinear dynamic behavior, which is often modeled using ordinary differential equations (ODEs). A common approach to constructing an ODE model of an observed biological system is to decompose the modeling process in two tasks [1,2]. The first task, referred to as *structure identification *and often solved by a modeling expert, is to specify the model structure, i.e., the functional form of the model ODEs. The second task of determining appropriate values for the model constant parameters, based on observations and measurements, is referred to as *parameter estimation*.

Due to the highly nonlinear dynamics and the limited measurability of biological systems, the parameter estimation task is challenging and computationally expensive. Most parameter estimation tasks in system biology are multi-modal, i.e., have many local optima that prohibit the use of local search methods. Furthermore, the models are often high-dimensional, making the parameter estimation task computationally complex. Finally, the measurability of systems in cell and molecular biology is highly limited. Many system variables are not directly observable. For the few ones that can be measured, measured data are noisy and taken at a coarse time resolution. All these constraints, combined with the complex dynamic of the considered models, can lead to identifiability problems, i.e, the impossibility of unique estimation of the unknown model parameters, making the parameter estimation an even harder optimization task [3].

There are two broad classes of approaches to the parameter estimation task: the *frequentist *(referred to as the "classical") approach and the *Bayesian *(probabilistic) approach [4,5]. The most representative approach of the first class is *maximum-likelihood *estimation (ML), according to which the most likely parameter values are the ones that maximize the probability (likelihood) of observing the given data. A special case of maximum-likelihood estimation, based on the assumption of independent and normally distributed errors in the experimental data, leads to the well-known approach of *least-squares *estimation (LS). Unlike ML estimation, which does not need any external information about the parameters, Bayesian estimation treats the parameters to be estimated as random variables, with a prior distribution representing the knowledge about the parameter values before taking the data into account. According to the information that the end-user has to provide, LS estimation is the simplest approach, while Bayesian approaches are the most complex ones [4,6].

Representative methods from both classes of approaches are commonly used for parameter estimation in the field of system biology [7-13]. It is difficult to argue in favor of one class of approaches against the other in a general manner [5], since both have shown advantages in specific situations. On one hand, Bayesian approaches can elegantly treat the uncertainty in parameter values and model structure in a uniform manner. On the other hand, frequentist approaches (such as the ones considered in this paper) can be effectively used for high-dimensional models with large numbers of parameters. We thus approach the parameter estimation task from the frequentist point of view and use least-squares estimation.

Related work using least-squares methods for parameter estimation in system biology [7-10] has shown that a proper way to address the above mentioned challenges in parameter estimation is to employ global optimization (GO) methods, especially stochastic GO methods and hybrid methods (that combine GO and local search methods). The advantage of stochastic methods is in their ability to handle black-box optimization problems and to converge relatively quickly to the vicinity of global optima. In this context, we employ three bio-inspired meta-heuristic global optimization methods.

We address the task of estimating the parameters of a nonlinear ODE model of endocytosis, more specifically of the maturation of endosomes, which are membrane-bound intracellular compartments used to transport and disintegrate external cargo. The model focuses on a key endocytotic regulatory system that switches from cargo transport in early endosomes to cargo disintegration in mature endosomes [14,15]. The regulatory system is based on the process of conversion of Rab5 domain proteins to Rab7 domain proteins. Using both a theoretical and an experimental approach to model this process, Del Conte-Zerial *et al*. [16] show that a cut-out switch ODE model provides the best fit to the biological observations, which show a rapid transition from the state with high Rab5 and low Rab7 concentrations in early endosomes to the inverse state of low Rab5 and high Rab7 concentrations in mature endosomes. The task of modeling the Rab5-to-Rab7 conversion has all the challenging properties mentioned above. Most notably, we have to cope with the limited measurability of the concentrations of Rab5 and Rab7 domain proteins in the endosome, since the different (i.e., active and passive) states of these proteins can not be distinguished in the measurement process.

In this paper, we study the effect of this kind of limited observability of the system dynamics on the complexity of the parameter estimation task, as well as the applicability and performance of four different optimization methods in this context. In order to do so, we define four different observation scenarios and generate artificial (pseudo-experimental) data for each of them. The scenarios cover a wide range of situations, from the simplest one of complete observability, where the concentrations of all protein states are assumed to be directly measurable, to the most complex (and realistic) scenario, where the observations are limited to the total concentrations of proteins in all their different states. We test the performance of the selected optimization methods in the different observation scenarios and compare the ability of different methods to cope with them. A final set of experiments, based on real-experimental data, are performed in order to check the validity of the results obtained on artificial data. More specifically, we test the methods' performance on measured data obtained through real-world biological experiments that corresponds to the most complex observation scenario described above.

Our study includes four optimization methods: the differential ant-stigmergy algorithm (DASA), our own, recently developed meta-heuristic method for global optimization [17,18]; particle swarm optimization (PSO), another bio-inspired meta-heuristic based on the idea of swarm intelligence [19]; differential evolution (DE), a well-known meta-heuristic method for global optimization based on the natural evolution concept [20,21]; and the local-search derivative-based algorithm 717 (A717) [22] updated with random restarts to cope with the multiple local optima problem. While PSO, DE, and A717 have already been applied to the task of parameter estimation in ODE models, DASA is tested in this context for the first time. Our initial study [23] has shown that DASA performs competitively to DE, and significantly better than A717 with random restarts. In this paper, we extend the preliminary tests with experiments in different, more realistic, observation scenarios, using both artificial data and laboratory measurements. We also investigate the practical identifiability of the model parameters under the different observation scenarios.

### Parameter estimation in ODE models

The task of parameter estimation in ODE models can be formalized as follows. Given a model structure *m*(*c*), which includes a set of adjustable parameters *c *= {*c*_1_, ..., *c_D_*}, and a set of observed data *d*, the task is to find the optimal values *c*^opt ^of *c *that lead to a model that reproduces the observed data *d *in the best possible way. Parameter estimation is usually approached as an optimization task of minimizing an objective function that measures the goodness of fit of the model simulation to the observed data.

#### Nonlinear least-squares estimation

Among different suggested objective functions measuring the goodness of fit, the maximum-likelihood estimator [4,24] introduced by R. A. Fisher in 1912, maximizes the probability of observing the given data *d *if the model *m*(*c*^opt^) is chosen. The likelihood function depends on the probability of the measurements in *d*. Assuming that the measurements follow independent normal distributions with a constant (unknown) variance, the maximum-likelihood parameter estimation maps into a nonlinear least-squares estimation of the parameters, which minimizes the *sum of squared errors *(SSE). For the observed data *d *= {*Y_i _*[*j*],1 ≤ *i *≤ *M*, 1 ≤ *j *≤ *N*}, SSE is defined as

(1) $$\text{SSE}\;\left(m\left(c\right)\right)=\overset{M}{\underset{i=1}{\sum }}\overset{N}{\underset{j=1}{\sum }}{\left(Y_{i}\left[j\right]-{Ŷ}_{i}\left[j\right]\right)}^{2},$$

where *Y_i_*[*j*] is the value of the *i*^th ^measured output at the *j*^th ^time point, *M *is the number of measured outputs, *N *is the number of samples per observed output, and  is the value of the *i*^th ^output at the *j*^th ^time point, predicted by the model *m*(*c*).

#### ODE models and observability

A model based on ODEs defines the temporal changes of a set of *system *(also referred as to endogenous) variables *S *as a function of the variables *S *and a set of *exogenous *variables *E*. The exogenous variables *E *are observed variables on which the model depends and appear on the right-hand side of the ODEs only, while the system variables *S *are dependent variables, the behavior of which is being modeled, and appear both on the left-hand side and the right-hand side of the ODEs. The ODE model of the observed system takes the form

(2) $$\frac{d}{dt}S=F\left(S,\;E,\;c\right),$$

where *t *represents time,  represents the time derivatives of the system variables *S*, *F *asserts the structure of the ODEs, and *c *is the set of model parameters. Such an ODE model, given the values of *S *at the initial time point *t*_0_, *S*(*t*_0_) and the values of the exogenous variables *E*(*t*) on the observed time interval [*t*_0_, *t*_*N*-1_], can be simulated to obtain the values of the system variables *S *in the time interval (*t*_0_, *t*_*N*-1_].

An analytical solution for complex nonlinear ODE integration problems does not exist in general: One has to apply numerical approximation methods for ODE integration. To this end, we use the CVODE package, a general-purpose ODE solver that uses the adaptive-step Adams-Moulton and backward differentiation method for integration [25].

Note that the ODE model captures the behavior of the system variables *S*, which do not directly correspond to the output (observed) variables *Y*. In general, the output *Y *of the ODE model at a time point *t *is modeled as

(3) Ŷ(t)=G(S(t),E(t)).

In the simplest observation scenario, the values of all the system variables are directly observed (measured), i.e., . However, in most real-world cases, especially in biology, we can not observe the values of all the system variables directly. This is mostly due to the physical limitations of the measurement methodology. Thus, when modeling biological systems, we deal with a variety of observation scenarios with different complexities. One possible scenario corresponds to situations where only some of the system variables are directly observed, i.e., . In an alternative, more complex observation scenario, *G *is a linear function, denoting, for example, that only the sum of the system variables can be directly observed, i.e., . Finally, the most complex observation scenarios involve arbitrary non-linear functions *G*. The complexity of the observation scenario can enormously influence the performance of parameter estimation methods, as we have to reconstruct the complete model based on incomplete observations. In this paper, we examine the influence of the observation scenario on the performance of parameter estimation methods in the context of modeling endocytosis.

## Methods

### Optimization methods

This section describes the optimization approaches used to solve the nonlinear parameter estimation task for the Rab5-to-Rab7 conversion model. We address the task using a recently-developed swarm-based meta-heuristic differential ant-stigmergy algorithm (DASA), motivated by the fact that DASA has shown promising results in solving large scale continuous global optimization problems, but has not been applied to the challenging task of parameter estimation in nonlinear ODE models. In addition, we use two well established meta-heuristics for global optimization, i.e., particle swarm optimization (PSO) and differential evolution (DE), as well as the derivative-based algorithm 717 (A717), essentially designed for nonlinear least-squares estimation. Below we provide a description of each of the four methods. We also specify the specific parameter settings in all methods as used in our experimental evaluation. The used parameter settings were selected by Sobol'-sampling-based parameter tuning [26].

#### The differential ant-stigmergy algorithm

The differential ant-stigmergy algorithm (DASA) was initially proposed in 2006 by Korošec [17]. It is a version of an ant colony optimization (ACO) meta-heuristic [27], designed to successfully cope with high-dimensional numerical optimization problems. The rationale behind the algorithm is in memorizing the "move" in the search space that improves the current best solution and using it in further search. The algorithm uses pheromones as a means of communication between ants (a case of stigmergy), combined with a graph representation of the search space. The DASA approach that we present here is slightly different from the initial DASA version [17]: It is described in detail by Korošec *et al*. [18], where a reference to the available source code [28] is given. The later was used in our experimental evaluation. 

First, the DASA approach transforms the *D*-dimensional optimization problem into a graph-search problem. The differential graph used in DASA is a directed acyclic graph obtained by fine-grained discretization of the continuous parameters' differences (offsets). The graph has *D *layers with vertices, where each layer corresponds to a single parameter. Each vertex of the graph corresponds to a parameter offset value that defines a change from the current parameter value to the parameter value in the next search iteration. Furthermore, each vertex in a given layer is connected with all vertices in the next layer. The set of possible vertices (discretized offset values) for each parameter depends on the parameter's range, the discretization base *b*, and the maximal precision of the parameters *ϵ*, which defines the minimal possible offset value. Ants use these parameters' offsets to navigate trough the search space. At each search iteration, a single ant positioned at layer *ℓ *moves to a specific vertex in the next layer *ℓ *+ 1, according to the amount of pheromone deposited in the graph vertices belonging to the (*ℓ *+ 1)-th layer: the probability of a specific vertex to be chosen is proportional to the amount of pheromone deposited in the vertex.

 Second, DASA performs pheromone-based search that involves best-solution-dependent pheromone distribution. The amount of pheromone is distributed over the graph vertices according to the Cauchy probability density function (PDF) [26]. DASA maintains a separate Cauchy PDF for each parameter. Initially, all Cauchy PDFs are identically defined by a location offset *l *= 0 and a scaling factor *s *= 1. As the search process progresses, the shape of the Cauchy PDFs changes: PDFs shrink as *s *decreases and stretch as *s *increases, while the location offsets *l *move towards the offsets associated with the better solutions. The search strategy is guided by three user-defined real positive factors: the global scale increase factor *s*_+_, the global scale decrease factor *s*_-_, and the pheromone evaporation factor *ρ*. In general, these three factors define the balance between exploration and exploitation in the search space. They are used to calculate the values of the scaling factor, *s *= *f*(*s*_+_, *s*_-_, *ρ*), and consequently influence the dispersion of the pheromone and the moves of the ants.

**Figure 1:**
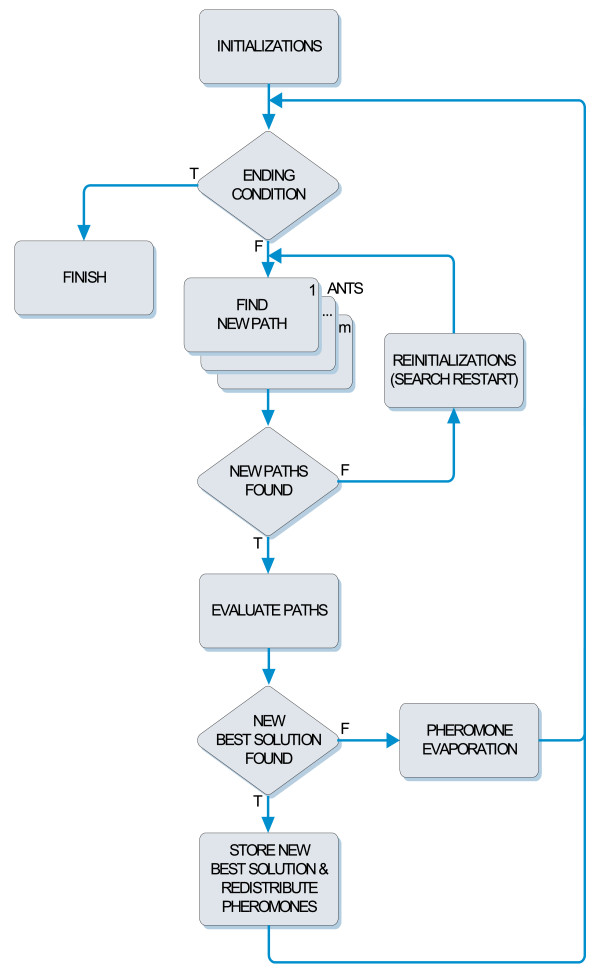
**The differential ant-stigmergy algorithm (DASA)**. High-level block-diagram representation of the DASA method.

The main loop of the DASA method, visualized in Figure 1, consists of an iterative improvement of a temporary-best solution, performed by searching (constructing) appropriate offset paths in the differential graph. The search is carried out by *m *ants, all of which move simultaneously from a starting vertex to the ending vertex at the last level, resulting in *m *constructed paths. Based on the found paths, DASA generates and evaluates *m *new candidate solutions. The best among the *m *evaluated solutions is preserved as a current-best solution. If the current-best solution is better than the temporary-best solution, the later is replaced, while the pheromone amount is redistributed along the path corresponding to the temporary-best solution and the scale factor is accordingly modified to improve the convergence. If there is no improvement over the temporary-best solution, then the pheromone distributions stay centered along the path corresponding to the temporary-best solution, while their shape shrinks in order to enhance the exploitation of the search space. If for some predetermined number of tries (in this case *D*^2 ^for all ants) all the ants only find paths composed of zero-valued offsets, the search process is restarted by randomly selecting a new temporary-best solution and reinitializing the pheromone distributions. Related to this, DASA keeps information about a globally best solution, called global-best solution. This solution is the best over all restarted searches, while the temporary-best solution is the best solution found within one search (restart).

#### Particle swarm optimization

Particle swarm optimization (PSO) is a stochastic population-based optimization technique developed by Eberhart and Kennedy in 1995 [19,29], inspired by the concept of social behavior of biological organisms, e.g., bird flocking or fish schooling [30]. A PSO algorithm maintains a swarm of particles, corresponding to a population of candidate solutions. Every particle moves ("flies") in the search space, adjusting its position and velocity according to its own experience and the social experience obtained by social interaction with the neighboring particles. PSO evidently shares similarities with evolutionary algorithms, developed on the basis of the Darwinian theory of evolution: both are inspired from natural phenomena and both maintain a population of candidate solutions and iteratively update (transform) the population using a variety of operators in order to find the optimal solution. However, PSO does not have selection, crossover or mutation operators: The main driving force of the swarm is the social interaction implicitly encoded in the social network structure. The social network structure is determined by the neighborhood of each particle, within which the particles can communicate by exchanging information about their success in the search space.

The basic PSO method initializes the swarm with *S *uniformly-random positioned particles in the search space. The search for the optimal solution proceeds in iterations. In every iteration, the current position (at time *t*) of the particle *x*(*t*) is incrementally updated with the new velocity *υ*(*t *+ 1) (i.e., *x*(*t *+ 1) = *x*(*t*) + *υ*(*t *+ 1)), which on the other hand is updated by using two sources of information. The first one, called cognitive component, reflects the experimental knowledge of the particle, which is its best position *x_p_*(*t*) found so far. The second one, called social component, reflects the local knowledge of the search space obtained from the particle's neighborhood with size *K *and is represented by *x_n_*(*t*), the best position found by the neighborhood of particles. The resulting formula for updating the velocity is then *υ*(*t *+ 1) = *υ*(*t*) + *c*_1_*r*_1_(*x_p_*(*t*) - *x*(*t*)) + *c*_2_*r*_2_(*x_n_*(*t*) - *x*(*t*))), where *c*_1 _and *c*_2_, called acceleration coefficients, are positive real values that balance the influence of the cognitive and the social component, while *r*_1 _and *r*_2 _are random factors uniformly sampled from the unit interval that introduce a stochastic component in the search.

The particular version of PSO used in our experimental evaluation is a standard variation of the basic PSO (the implementation is available online [31]), which includes only one acceleration coefficient *c *and an additional mechanism to control the exploration and exploitation in the search space via the parameter *w*, called inertia weight. The inertia weight basically controls the influence of the previous search direction on the new velocity. At each iteration, each particle chooses a few particles to be its informants, selects the best one from this set (neighborhood), and takes into account the information given by the chosen particle (the best informant). If there is no particle better than itself, either the informant stays the same (default setting), or the informant is chosen randomly (optional setting). The velocity is updated according to the expression *v*(*t *+ 1) = *wv*(*t*) + *g*(*t*) -*x*(*t*) + *H *(*g*(*t*), ||*g*(*t*) - *x*(*t*)||), where the function *H *returns a random point inside the hypersphere with center of gravity *g*(*t*) and radius ||*g*(*t*) - *x*(*t*)||. The center of gravity is defined as .

#### Differential evolution

Differential evolution (DE) is a simple and efficient population-based heuristic for optimizing real-valued multi-modal functions, introduced by Storn and Price in the 1990s [21,32]. It belongs to the class of evolutionary algorithms (EA) based on the idea of simulating the natural evolution of a population *P *of individuals (candidate solutions) via the processes of selection, mutation and crossover.

The main difference between traditional EA and DE is in the reproduction step, where for every candidate individual *x_c _*an offspring *u *is created by using a mutated individual *υ*. The latter is obtained by a simple arithmetic (differential) mutation operation over a set of parents (e.g., *x*_1_, *x*_2_, *x*_3_) selected at random or by quality, based on one difference vector, i.e., *υ *= *x*_1 _+ *F*·(*x*_2 _- *x*_3_). The rate at which the population evolves can be controlled by a scale (mutation) factor *F*, a user-defined positive real number from the interval [0, 2]. To complement the differential mutation strategy, DE employs uniform crossover (also known as discrete recombination) over the candidate and mutated individual in order to generate the offspring *u*. A user-specified crossover factor *CR *∈ [0, 1] is used to control the fraction of parameter values copied from the mutated individual to the offspring. Finally, the offspring is evaluated and if its fitness (objective function) is better, it replaces the corresponding candidate individual in the population. Depending on the specific mutation and crossover procedure, one can chose among several DE strategies identified using the name format "DE/*x/y/z*". In the name format, *x *represents a string denoting the solution to be perturbed: i) a solution randomly chosen from the population (*x *="rand"); ii) the current best solution (*x *= "best"); or iii) a solution based on the candidate solution combined with a difference vector towards the current best individual (*x *= "rand-to-best"), i.e., *x_c _*+ *F*·(*x_best _*- *x_c_*). Further, *y *represents the number of difference vectors considered for perturbation, while *z *stands for the type of crossover being used that can be exponential (*z *= "exp") or binomial (*z *= "bin").

The implementation used in our experimental evaluation is based on the implementation of the DE algorithm described in the technical report by Storn and Price [32], available online [33]. It includes 10 search strategies, *STR*, enumerated from one to 10 in the following order: 1 - "DE/best/1/exp", 2 - "DE/rand/1/exp", 3 - "DE/rand-to-best/1/exp", 4 - "DE/best/2/exp", 5 - "DE/rand/2/exp", 6 - "DE/best/1/bin", 7 - "DE/rand/1/bin", 8 - "DE/rand-to-best/1/bin", 9 - "DE/best/2/bin", and 10 - "DE/rand/2/bin". As the original code does not check whether the newly generated solutions are allowed, i.e., lie within the prescribed parameter ranges, we slightly modified the code: if the new solution is outside the specified bounds, it is set to the closest range limit.

#### Algorithm 717

Algorithm 717 (A717) is a set of modules for solving the parameter estimation problem in nonlinear regression models like nonlinear least-squares, maximum-likelihood and some robust fitting problems [22]. The basic method is a generalization of NL2SOL - an adaptive nonlinear least-squares algorithm [34], which uses a model/trust-region technique for computing trial steps along with an adaptive choice for the Hessian model. In fact, NL2SOL is a variation of the Newton's method (augmented Gauss-Newton method), in which a part of the Hessian is computed exactly and a part is approximated by a secant (quasi-Newton) updating method. Thus, the algorithm sometimes reduces to the Gauss-Newton or the Levenberg-Marquardt method.

In order to promote convergence from poor starting guesses, the algorithm implements the idea of having a local quadratic model *q_i _*of the objective function *f *at the current best solution *c_i _*and an estimate of an ellipsoidal region centered at *c_i _*in which *q_i _*is trusted to represent *f*. So the next point, *c*_*i*+1_, or the next trial step, is chosen to approximately minimize *q_i _*on the ellipsoidal trust-region. The information obtained for *f *at *c*_*i*+1 _is used for model updating and also to resize and reshape the trust-region.

Among the modules, we can chose the ones for unconstrained optimization, or the ones that use simple bound constraints on the parameters. Furthermore, we can choose between modules that involve approximate computation of the needed derivatives by finite differences, and modules that expect the derivatives of the objective function to be provided by the routine that calls them.

In this work, we used the original implementation of A717 as available online [35]. Since A717 is not a global search algorithm, we wrapped the original procedure in a loop of restarts with randomly chosen initial points, providing in a way a simple global search. The number of restarts was set to 20000 (25 evaluations per restart) to result in a number of function evaluations comparable to the number used for the other three methods.

#### Parameter settings

In the text above, we described the optimization methods that will be used for parameter estimation in the endocytosis model. Among these, the meta-heuristic approaches have many parameters that guide the search and consequently influence the methods' performance. To obtain the best possible performance on a given problem, one should consider a task specific tuning of the parameter setting for the optimization method used (see, e.g., the study by Daeger *et al*. [10] in the domain of system biology). Determining the optimal parameter setting is an optimization task in itself, which is extremely computationally expensive.

There are two common approaches for choosing parameters values [36]: *parameter tuning *and *parameter control*. The first approach selects the parameter settings before running the optimization method (and they remain fixed while performing the optimization). The second approach optimizes the method's parameters along with the problem's parameters.

A detailed discussion and survey of parameter tuning methods is given by Eiben and Smit [36], who identify *sampling *methods as one type of parameter tuning methods. Sampling methods reduce the search effort by decreasing the number of investigated parameter settings as compared to the full factorial design. Two widely used sampling methods are Latin-squares and Taguchi orthogonal arrays (appropriate references are given by Eiben and Smit [36]).

In this paper, parameter tuning for the meta-heuristic optimization methods was performed with a sampling method based on Sobol' sequences, introduced by Sobol' in 1967 [26]. Sobol' sequences, sampled from the *d*-dimensional unit search space, are quasi-random sequences of *d*-tuples that are more uniformly distributed than uncorrelated random sequences of *d*-tuples. These sequences are neither random nor pseudo-random, as they are cleverly generated not to be serially uncorrelated, but instead to take into account which tuples in the search space have already been sampled. For a detailed explanation and overview of the schemas for generating Sobol' sequences, we refer to Press *et al*. [26]. The particular implementation of Sobol' sampling used in this paper is based on the Gray code order and is available online [37].

The DE method has only four parameters, while DASA and PSO have more: Consequently we chose only four parameters per single method to be tuned. For DASA, we chose the three real-valued parameters that directly influence the search heuristic (*s*_+_, *s*_-_, and *ρ*) and the number of ants (*m*), while for PSO, we chose the size of the swarm (*S*), the size of the neighborhood (*K*), the inertia weight (*w*), and the acceleration coefficient (*c*). The number of sampled parameter settings (4-tuples) per method was 2000. Due to the stochastic nature of the methods, every parameter setting was used for optimization of the endocytosis model in a multiple-run experimental evaluation that included half a million objective function evaluations. The number of runs was set to eight. The optimal performing parameter setting was chosen based on the median best performance over all runs. A common approach is to use the mean best performance, but we took the median in order to avoid the problems that the mean has when observing large variance in the objective function values across the runs.

The parameters of the three meta-heuristics methods chosen for Sobol'-sampling-based parameter tuning and their ranges are summarized in Table 1. In the same table, we report the resulting (tuned) parameter settings for the three optimization methods. Note that the Sobol' sampling approach generates number on the unit interval: In order to obtain the parameter settings, we had to map that value on the predefined range of parameter values. For the integer-valued parameters, the mapped value was rounded to the closest integer value. An additional note concerns the upper bound of the parameter *K*, denoting the size of the neighborhood in the PSO method, which can not be larger than the value *S *- 1. Its value was mapped according to the chosen value of the size of the swarm *S*. In a similar way, the upper bound of the global scale decrease factor *s*_ is limited by the value of the evaporation factor *ρ *in the DASA method. Finally, note that the parameter tuning was performed on the complete observation scenario using noise-free artificial data. The same parameter settings were then used across all scenarios and all datasets. The parameter settings used in our experimental evaluation are given as follows.

**Table 1:** Setup and results of Sobol'-sampling-based parameter tuning of optimization methods.

	DASA				PSO				DE			
			
Parameter	*m*	*Ρ*	*s*+	*s*-	*S*	*K*	*w*	*c*	*P*	*ST R*	*F*	*CR*
Lower	4	0	0	0	4	1	0	1	6	1	0	0
Upper	200	1	1	*ρ*	200	*S*-1	1	4	200	10	2	1

Tuned	144	0.036	0.573	0.01	155	89	0.762	1.037	81	8	0.942	0.915

The table includes the search ranges (their lower and upper bound) for each of the four parameters of each of the three different meta-heuristic optimization methods that were tuned. We used the Sobol' sampling procedure with the number of sampling points set to 2000. The resulting vector of method's parameters was chosen as the one that showed best median performances (according to the SSE metric) in the multiple-run experiments among the 2000 sampled parameter settings. A single experiment included eight runs, each performed with half a million of objective function evaluations. The parameter tuning was performed on the complete observation scenario using noise-free artificial data.

##### DASA setup

The discretization base is set to 10, the maximum parameter precision is set to 10^-15^, the number of ants is set to 144, the global scale increase factor to 0.575, the global scale decrease factor to 0.01, and the pheromone evaporation factor to 0.036.

##### PSO setup

A variable random topology was chosen, the particle swarm size was set to 155, the neighborhood size to 89, the inertia weight to 0.762, and the acceleration coefficient to 1.037. In addition, default settings were used for the remaining parameters related to advanced options not included in the standard PSO method.

##### DE setup

The chosen strategy was "DE/rand-to-best/1/bin", the population size was set to 81, the weight factor to 0.915, and the crossover factor to 0.942.

### Comparison methodology

To guarantee a fair comparison of the three optimization methods, we ran each method 25 times allowing half a million of evaluations of the objective function per single run. We used a number of performance evaluation metrics to compare the utility of the three optimization methods for parameter estimation; the reported method performance is the average/median performance over all 25 runs. While the first quality measure is about the convergence rate of the optimization *methods*, the others focus on the quality of the obtained *models*.

*Convergence curves *are commonly used for visualizing the convergence rates of optimization methods. They show the change of the value of the objective function with the increasing number of objective function evaluations. Each curve in our paper depicts the change of the objective function value averaged over 25 runs: The convergence curves are plotted on log-log plots, with a logarithmic scale for both axes in order to be able to capture the convergence trend over a wide range of values.

*Root mean squared error *(RMSE) measures the difference between output values predicted by the model  and the observed values of the output variables *Y*.

(4) $$\begin{array}{lll} \text{RMSE} & =\sqrt{\frac{1}{N}\overset{M}{\underset{i=1}{\sum }}\overset{N}{\underset{j=1}{\sum }}{\left(Y_{i}\left[j\right]-{Ŷ}_{i}\left[j\right]\right)}^{2}}\; & \text{(1)}\\ & =\sqrt{\frac{1}{N}\text{SSE}\left(m\left(c\right)\right)}\; & \text{(2)}\\ & \; & \text{(3)} \end{array}$$

The division by the number of data points and square root in RMSE make its measurement units and scale comparable to the ones of the observed output variables. This is in contrast with the SSE measure defined with Eq. (1). Finally, note that better models have smaller values of RMSE.

As defined above, the RMSE quality metric measures the degree-of-fit between simulated model output and observed system output. However, reconstruction of system dynamics goes beyond reconstructing output; ultimately, modeling is about capturing the complete (also unobserved) system dynamics. To measure this aspect of reconstruction quality, we have to measure the degree-of-fit between simulated and observed values of the system variables. Although this is impossible in real cases where system variables can not be directly observed, experiments with artificial data allow us to measure this aspect of model quality. In this context, we use an additional model quality metric when comparing the methods in the case of artificial data.

The *root mean squared error of the completely simulated model *(RMSEm) is defined as

(5) $$\text{RMSEm}=\sqrt{\frac{1}{N}\overset{4}{\underset{i=1}{\sum }}\overset{N}{\underset{j=1}{\sum }}{\left(S_{i}\left[j\right]-{Ŝ}_{i}\left[j\right]\right)}^{2}},$$

where *S_i_*[*j*] and  are the values of the system variables from the reference model and the estimated (predicted) model, respectively. This metric will allow us to test whether the good quality of the model in terms of outputs is related to the ability of the model to capture the unobserved system dynamics. To represent the distributions of the quality measure values over the 25 runs of a specific optimization method on a specific observation scenario and data set, we used *box-and-whisker diagrams (boxplots)*. They provide a convenient graphical representation of the dispersion, skewness, and outliers in a single given data sample, but also enable a visual comparison of different data samples. The top and bottom edge of the box in a boxplot represent the 25^th ^and 75^th ^percentiles of the sample, respectively; in consequence, the box height corresponds to the *interquartile range *(IQR). The line in the middle of the box corresponds to the sample median. The sample mean is represented with a diamond. The "whiskers", i.e., the two lines extending above and below the box, represent the sample range. The maximal length of the whiskers is set to 1.5·IQR. Data points above and below the whiskers' end points correspond to the sample outliers and are represented with "+" markers. Finally, the notches of the boxes represent the variability of the median in the sample. The width of a notch is computed so that boxplots whose notches do not overlap have medians that are significantly different at the 0.05 significance level, assuming a normal data distribution. The boxplots presented in this paper were generated using the MATLAB statistical toolbox [38].

*Statistical significance testing *was performed in order to assess the obtained differences in performance between the four optimization methods. We used the post-hoc multiple comparison Holm test [39], according to which we first rank the compared methods based on their performances averaged over all test problems and assign a score *r_i _*for *i *= 0, ..., *N_m _*- 1, where *N_m _*is the number of methods being compared and *i *is the appropriate rank (*i *= 0 corresponds to the best ranked method, while *i *= *N_m _*- 1 to the worst ranked method). Second, we select the method with the best score (lowest rank), *r*_0 _and calculate the values

(6) $$z_{i}=\frac{r_{0}-r_{i}}{\sqrt{\frac{N_{m}\left(N_{m}+1\right)}{\left(6N_{tp}\right)}}},$$

where *N_tp _*is the number of considered test problems for each method. Finally, we calculate the cumulative normal distribution values *p_i _*corresponding to *z_i _*and compare them with the corresponding *α*/*i *values where *α *is the significance level, set to 0.05 in our case. We report the *z_i_*, *p_i_*, and *α*/*i *values in a table, where each row corresponds to one of the methods. The best ranking method, used as reference method, is excluded from the table. Based on these values, we can make a decision about the null-hypothesis that "there is no difference in performance between this and the best ranking method".

### Practical parameter identifiability

The problem of uniqueness of the estimated parameters in a given model is related to the issue of parameter identifiability. We can distinguish between structural and practical identifiability. Structural identifiability is a theoretical property of the model structure, depending only on the model input (stimulation function) and output (observation function): It is not related to the specific values of the model parameters. The parameters of a given model are structurally globally identifiable, if they can be uniquely estimated from the designed experiment under the ideal conditions of noise-free observations and error-free model structure [40]. If the model is not structurally identifiable, one should consider reformulating the model.

Even when we deal with a structurally identifiable model, it can still happen that the parameters can not be uniquely identified from the available experimental data. In this case, we experience a practical identifiability problem, related to the amount and quality of available experimental data. Practical identifiability analysis can also help us to assess the uncertainty of the parameter estimates and to compare possible experimental designs without performing experiments. Parameter uncertainties (confidence intervals) may be computed by using the Fisher information matrix (FIM) or a Monte Carlo-based approach. Details and further references on this topic are given by Balsa-Canto and Banga [3].

We are going to assess the practical identifiability of the parameters in the endocytosis model using the Monte Carlo-based sampling approach [3,41]. The approach estimates the expected uncertainties of the parameters by re-applying the parameter estimation method to a large number of replicate datasets generated by using different realizations of the chosen experimental noise. In this way, we generate a cloud of parameter estimates that represents the confidence region. Based on this cloud of solutions, we can obtain the distribution (represented by histograms) of values for each uncertain parameter (as well as its mean value and standard deviation) and make correlation analysis to determine the most correlated parameters. In contrast to the FIM-based approach, that assumes a linearization of the model, the Monte Carlo approach estimates are reliable also for highly non-linear models or very large parameter uncertainties. The generality of the Monte Carlo approach, however, comes at a high computational cost. For the specific task of modeling endocytosis, we generated one thousand datasets by simulating the model with the reference parameter values and adding Gaussian noise. Given a percentage of a relative noise level *s*, we calculated the noisy value as *Y*_noisy _= *Y *(1 + *s*·*N *(0, 1)), considering a noise level of 20%. We estimated the parameters of the model using these datasets, and collected the estimates for outlier examination and further statistical analysis. We followed the same procedure as described by Joshi *et al*. [41], according to which outliers are data points that do not belong to the interval [*Q*1 - 1.5(*Q*3 - *Q*1), *Q*3 + 1.5(*Q*3 - *Q*1)], where *Q*1 and *Q*3 represent the 25^th ^and 75^th ^percentiles of the sample, respectively. The detected outliers are removed: More precisely, the new (reduced) sample includes only the estimates obtained from those datasets that did not produce any outlier over all parameters. The distributions of the parameters are presented with histograms, including the corresponding 95% confidence intervals *CI*: These are calculated as the length of the interval between the 2.5^th ^and 97.5^th ^percentile of the sample. The width of the bins *h *for a single histogram is calculated according to the Freedman-Diaconis rule [42],

(7) $$h=\frac{2\;\cdot \;\text{IQR}}{\sqrt[{3}]{N_{s}}},$$

where IQR is the interquartile range of the sample and *N_s _*is the sample size.

Based on the outlier-free samples of parameter estimates, the correlation of two model parameters *c_i _*and *c_j _*in terms of a linear dependence is calculated based on the Pearson correlation coefficient [4] according to the formula

(8) $$R\left({\tilde{c}}_{i},{\tilde{c}}_{j}\right)=\frac{\sum_{k=1}^{N_{\text{s}}}{\tilde{c}}_{ik}{\tilde{c}}_{jk}-N_{s}\mu_{{\tilde{c}}_{i}}\mu_{{\tilde{c}}_{j}}}{N_{s}\sigma_{{\tilde{c}}_{i}}\sigma_{{\tilde{c}}_{j}}},$$

where  and  are the mean and the standard deviation of the vector  with estimates of the *c_i _*parameter, while  and  are the mean and the standard deviation of the vector  with estimates of the *c_j _*parameter. A correlation of 1 (or - 1) means perfect positive (or negative) linear relationship between the two parameters.

## Results and Discussion

### Endocytosis model

This work addresses the task of parameter estimation in a practically relevant model of endocytosis, i.e., the life-cycle of endosomes. Endosomes are membrane-bound intracellular components that typically encapsulate, transport, and disintegrate external cargo within cells. The model at hand focuses on the process of endosome maturation, representing it by a cut-out switch between the concentrations of Rab5 and Rab7 domain proteins [14,15]. The theoretical and experimental approach [16] undertaken to model the endocytosis rely on the mutual exclusiveness of the Rab5 and Rab7 domains. Early endosomes have with high Rab5 and low Rab7 concentrations, while late or mature endosomes have low Rab5 and high Rab7 concentrations. The transition from the early to the mature state is rapid.

To model the Rab5-to-Rab7 conversion, we distinguish between active and inactive (passive) states of the Rab5 and Rab7 domain proteins. Thus, the ODE model involves four system (endogenous) variables corresponding to the concentrations of Rab5 domain proteins in inactive (*r*_5_) and active state (*R*_5_) and Rab7 domain proteins in inactive (*r*_7_) and active state (*R*_7_), measured in mol/l. These four species (chemical compounds) are involved in ten different biochemical reactions *υ*_1_, ..., *υ*_10 _parameterized with eighteen constant parameters *c*_1_, ..., *c*_18 _corresponding to the kinetic rates of the reactions, leading to the following structure of the model ODEs:

(9) $$\begin{array}{c} \frac{d}{dt}r_{5}=v_{1}+v_{7}+v_{9}-v_{2}-v_{3}\\ \frac{d}{dt}R_{5}=v_{2}-v_{7}-v_{9}\\ \frac{d}{dt}r_{7}=v_{4}+v_{10}-v_{5}-v_{6}-v_{8}\\ \frac{d}{dt}R_{7}=v_{5}+v_{6}-v_{10}. \end{array}$$

Here, *υ*_1_, ..., *υ*_10 _denote the kinetic models of the corresponding biochemical reactions, given below:

(10) $$\begin{array}{c} v_{1}=c_{1}\\ v_{2}=\frac{c_{2}r_{5}\left(t\right)}{1+e^{\left(c_{3}-R_{5}\left(t\right)\right)c_{4}}}\frac{t}{100+t}\\ v_{3}=c_{5}r_{5}\left(t\right)\\ v_{4}=c_{6}\\ v_{5}=\frac{c_{7}r_{7}\left(t\right)R_{7}{\left(t\right)}^{c_{8}}}{c_{9}+R_{7}{\left(t\right)}^{c_{8}}}\\ v_{6}=\frac{c_{10}r_{7}\left(t\right)}{1+e^{\left(c_{11}-R_{5}\left(t\right)\right)c_{12}}}\\ v_{7}=\frac{c_{13}R_{5}\left(t\right)}{1+e^{\left(c_{14}-R_{7}\left(t\right)\right)c_{15}}}\\ v_{8}=c_{16}r_{7}\left(t\right)\\ v_{9}=c_{17}R_{5}\left(t\right)\\ v_{10}=c_{18}R_{7}\left(t\right). \end{array}$$

Note that all variables in the model are system variables, i.e., *S *= {*r*_5_, *R*_5_, *r*_7_, *R*_7_} and there are no exogenous variables, i.e., *E *= ∅.

**Figure 2:**
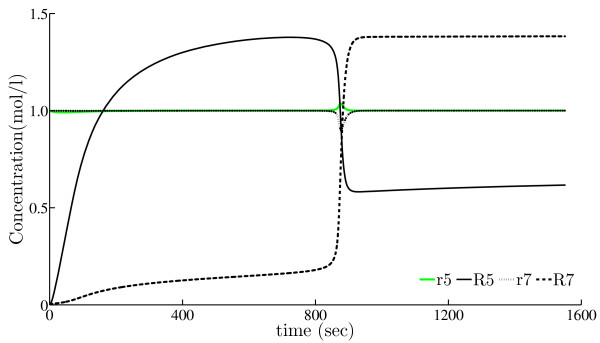
**Simulated behavior of the Rab5-to-Rab7 conversion model**. Simulation of the cut-out switch model of the conversion of Rab5 domain proteins to the Rab7 domain proteins in the regulatory system of endocytosis as proposed by Del Conte-Zerial *et al*. [16].

Figure 2 depicts the simulated behavior of the four system variables of the model in the time interval [0, 1551] seconds, with the parameters values set to

(11) $$\begin{array}{c} c_{1}=1,\;c_{2}=0.3\;c_{3}=0.1,\\ c_{4}=2.5,\;c_{5}=1,\;c_{6}=0.483,\\ c_{7}=0.21,\;c_{8}=3,\;c_{9}=0.1,\\ c_{10}=0.021,\;c_{11}=1,\;c_{12}=3,\\ c_{13}=0.31,\;c_{14}=0.3,\;c_{15}=3,\\ c_{16}=0.483,\;c_{17}=0.06,\;c_{18}=0.15, \end{array}$$

and initial values of the state variables set to

(12) $$\begin{array}{c} r_{5}\left(t_{0}\right)=r_{7}\left(t_{0}\right)=1\;\text{mo}1∕1,\\ R5\left(t_{0}\right)=R7\left(t_{0}\right)=0.001\;\text{mo}1∕1, \end{array}$$

as proposed by Del Conte-Zerial *et al*. [16]. Note that the behavior of the concentrations of the active-state proteins *R*_5 _and *R*_7 _follow the expected (rapid) cut-out switch from high Rab5 and low Rab7 to low Rab5 and high Rab7 concentrations, while the concentrations of the passive-state proteins *r*_5 _and *r*_7 _remain almost constant throughout the whole process, with a small but notable change at the transition point.

In sum, the task of parameter estimation in the Rab5-to-Rab7 cut-out switch model leads to a 22-dimensional continuous minimization problem with 18 dimensions corresponding to model parameters and four dimensions corresponding to the initial values of the four system variables. The objective function, *sum of squared errors *(SSE, as defined in Eq. (1)) between the observed and predicted values of the system output, is minimized with respect to the given data, subject to the structure of the ODEs of the Rab5-to-Rab7 conversion model (described by Eqs. (9) and (10)) and the following bound constraints on the values of the constant parameters and protein concentrations: *c_i _*∈ (0, 4] for 1 ≤ *i *≤ 18, *c_i _*∈ (0, 2] for 19 ≤ *i *≤ 22, *r*_5_(*t*) ≥ 0, *R*_5 _(*t*) ≥ 0, *r*_7 _(*t*) ≥ 0, and *R*_7 _(*t*) ≥ 0. To calculate the objective function, we perform ODE integration. Despite the fact that we use advanced adaptive-step integrators, for some parameter sets the ODE integration can fail, due to the discontinuities in the model dynamics: in that case or in the case of violation of the basic constraint about non-negative values for the simulated protein concentration, we simply discard the respective solution (by giving the objective function a very high real value).

In order to evaluate the performance of different parameter optimization methods on this task, we conducted experiments with artificial data, obtained by simulating the Rab5-to-Rab7 conversion model, and with real data from experimental measurements.

### Data

#### Artificial (pseudo-experimental) data

We generated the artificial data by simulating the ODE model from Eqs. (9)-(12) at 2781 equally spaced time points inside the interval [0, 1551] seconds. To obtain more realistic artificial data, we added a normal Gaussian noise *N*(0, 1) to the noise-free simulated data. Given the percentage of a relative noise level *s*, we calculate the noisy data as *Y*_noisy _= *Y *(1 + *s*·*N *(0, 1)). In our experiments, we use two noise levels of 5% and 20%. Note that the noise-free data correspond to the noise level of 0%.

#### Measured (real-experimental) data

In the second set of experiments, we used the real time-course measurements from Del Conte-Zerial *et al*. [16]. The measurements are taken following a complex procedure, where a number of endosomes were followed in three independent experiments for Rab5 (23 endosomes) and one for Rab7 (15 endosomes). Experimental data for different endosomes were manually aligned around the conversion point, scaled, and averaged at 10571 time points in the interval of [-5, 330] seconds, where time point 0 corresponds to the conversion point. Note however, that due to physical limitation of the measurement experiments, only the total concentrations of the active and inactive Rab5 and Rab7 domain proteins could be measured, leading to a complex observation scenario with the following two output variables *Y*_1_(*t*) = *r*_5_(*t*) + *R*_5_(*t*) and *Y*_2_(*t*) = *r*_7_(*t*) + *R*_7_(*t*).

### Observation scenarios

The limited measurability of the system variables in the real-world measurement scenario, described above, represents one of the most challenging properties of the parameter estimation task addressed in this paper. To evaluate the impact that the limited observability has on the difficulty of the optimization task (and consequently on the performance of different optimization methods), we define here four observation scenarios, ranging from the simplest one that assumes that all the system variables can be directly measured to the most complex one that corresponds to the limitations of the real measurement process described in the previous paragraph.

#### Complete observation (CO)

In this scenario, we assume that all the system variables are directly observed, meaning that the measurement process can identify the four concentrations of active and inactive states of the Rab5 and Rab7 proteins at each time point, i.e., *Y*_1_(*t*) = *r*_5_(*t*), *Y*_2_(*t*) = *R*_5_(*t*), *Y*_3_(*t*) = *r*_7_(*t*), and *Y*_4_(*t*) = *R*_7_(*t*).

#### Active-state protein concentration observation (AO)

Here, we assume that only concentrations of the active-state proteins can be observed, i.e., *Y*_1_(*t*) = *R*_5_(*t*) and *Y*_2_(*t*) = *R*_7_(*t*). This scenario is simpler than the real one. Since we can measure total (active-state and passive-state) protein concentration and the passive-state protein concentrations are expected to be constant most of the time (see Figure 2), this scenario is based on a reasonable assumption.

#### Total protein concentration observation (TO)

This scenario represents the real measurement process outlined above, where *Y*_1_(*t*) = *r*_5_(*t*) + *R*_5_(*t*) and *Y*_2_(*t*) = *r*_7_(*t*) + *R*_7_(*t*).

#### Neglecting passive-state protein concentration (NPO)

This is the scenario based on how the measurements are (visually) matched against model simulations by Del Conte-Zerial *et al*. [16]. In this case, we observe the total protein concentrations, i.e., *Y*_1_(*t*) = *r*_5_(*t*) + *R*_5_(*t*) and *Y*_2_(*t*) = *r*_7_(*t*) + *R*_7_(*t*), but we match them against concentrations of the active-state proteins predicted by the model, i.e.,  and . The rationale for this scenario is the same as for the second one (AO) and it is included here to match the procedure used by Del Conte-Zerial *et al*. [16].

### Parameter estimation with artificial data

Given the artificial data described above (obtained using the reference values of the constant parameters from Eq. (11), we can calculate the value of the objective function at the reference point for each noise level and observation scenario: These are reported in Table 2. Note that the value of the objective function at the reference point, when considering noise-free data, is zero, while in the case of noisy data it increases and becomes greater than zero. The exception to this rule is the NPO observation scenario used in Del Conte-Zerial *et al*. [16], where the authors assume that the concentrations of passive-state proteins can be neglected when fitting the data. This assumption is obviously implausible, since it leads to large values of the objective function, even in the case of noise-free data.

**Table 2:** Values of the quality metrics for the reference model.

Noise	Scenario	SSE	RMSE
	CO	0	0
0%	AO	0	0
	TO	0	0
	NPO	5549.839	1.413

	CO	2.653	0.031
5%	AO	1.289	0.022
	TO	2.591	0.031
	NPO	5556.486	1.414

	CO	42.447	0.124
20%	AO	20.627	0.086
	TO	41.452	0.122
	NPO	5607.516	1.420

The reference model was used for generation of the artificial data.

**Figure 3:**
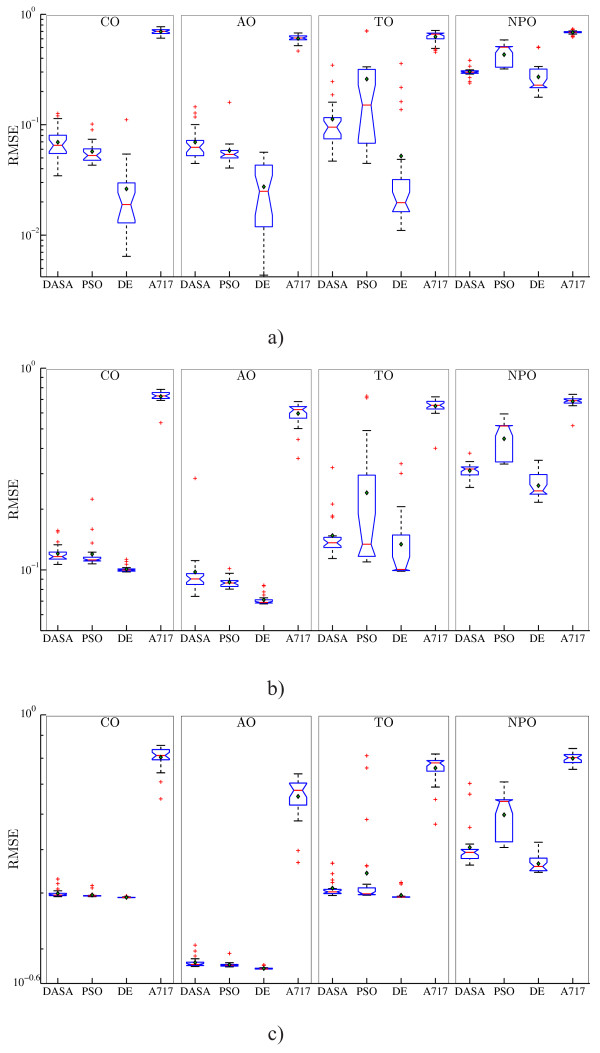
**RMSE performance of the models obtained by parameter estimation from artificial data**. Boxplots of the performance distributions of the four optimization methods (DASA, PSO, DE, and A717) in terms of the quality of the reconstructed output (RMSE), when considering four different observation scenarios (columns CO, AO, TO, and NPO) and three artificial datasets (rows): a) noise-free, *s *= 0%; b) noisy data, *s *= 5%; and c) noisy data, *s *= 20%. Due to the large differences in the order of magnitude, the RMSE values are plotted on a logarithmic scale.

Let us now consider the RMSE performance of the four parameter estimation methods (DASA, PSO, DE, and A717) on the artificial datasets with three levels of noise (0%, 5%, and 20%) under the four observation scenarios (CO, AO, TO, and NPO). Figure 3 summarizes the RMSE performance with boxplots over the 25 runs of each methods. The 12 graphs on the figure correspond to the four observation scenarios (in columns) and three artificial datasets (in rows), where each graph depicts the performance comparison of the four parameter estimation methods. The graphs show that the median performance of A717 is significantly worse than the performance of the three meta-heuristic methods. The comparison among the latter indicate that the median RMSE performance of DE is significantly better than the performance of DASA and PSO. These findings hold in all observation scenarios and at all noise levels. The performance comparison among different levels of noise shows a systematic decrease of the RMSE performance with the increasing noise level. The noise in the data affects the performance of all methods in all observation scenarios, but the magnitude of the effect differs. While we observe very large and remarkable differences in performance of the meta-heuristics methods in the noise-free case, there is much less difference in performance on the noisy datasets.

The comparison among observation scenarios shows that the CO and AO scenarios are very similar: they induce an identical ranking of the optimization methods in terms of performance at all noise levels. The rankings are slightly different (but still very similar) in the case of TO, and quite different in the implausible scenario (see the discussion above) of NPO. As the noise level increases, the AO scenario seems to become an easier task than the CO scenario leading to much better optimum values of RMSE, while the CO scenario becomes very similar to the TO scenario. In the NPO case, all four optimization methods overfit the observed output, leading to values of the objective function that are smaller than the value at the reference point from Table 2. Note also that the PSO and DE methods lead to a higher variance of RMSE across the different runs in the TO and NPO scenarios. Overall, the RMSE performance metric does not provide a clear and unified conclusion about the relative difficulty of the parameter estimation task under different observation scenarios.

**Figure 4:**
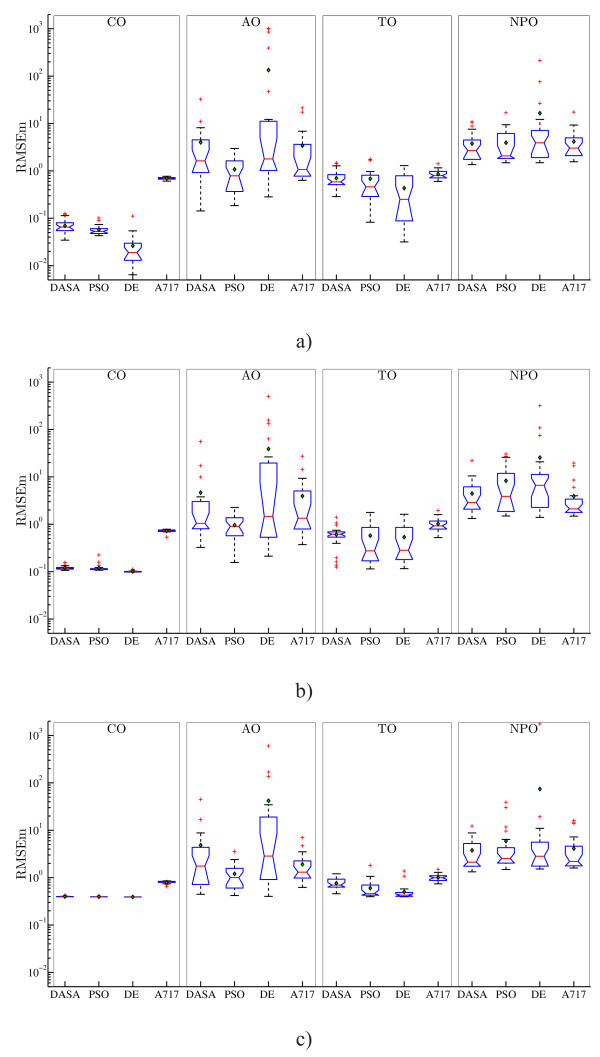
**RMSEm performance of the models obtained by parameter estimation from artificial data**. Boxplots of the performance distributions of the four optimization methods (DASA, PSO, DE, and A717) in terms of the quality of the complete model reconstruction (RMSEm), when considering four different observation scenarios (columns CO, AO, TO, and NPO) and three artificial datasets (rows): a) noise-free, *s *= 0%; b) noisy data, *s *= 5%; and c) noisy data, *s *= 20%. Due to the large differences in the order of magnitude, the RMSEm values are plotted on a logarithmic scale.

However, comparing the RMSEm performance, i.e., the quality of the complete model reconstruction, leads to much clearer conclusions about the relative difficulty of the four observation scenarios. Figure 4 summarizes the RMSEm performance. As one would expect, the easiest optimization tasks stem from the CO scenario, since in this scenario all the system variables are directly observed. However, note that the TO scenario, corresponding to the real biochemical measurement process, although complex (the observed outputs are linear combinations of the system variables) compares favorably to the other two scenarios in terms of complete system dynamics reconstruction. This can be explained by the fact that the observed outputs in the TO scenario carry more information about the system (they include both active and passive states of proteins) than the observed outputs (active-state proteins only) in the AO or NPO scenarios. The higher variance and evident outliers in the RMSEm values associated with the AO and NPO scenarios confirm that incomplete and/or misinterpreted measurements lead to more difficult optimization tasks. In the CO and TO scenarios, the performance in terms of RMSEm of the four optimization methods follows a similar pattern as the one for the output reconstruction (RMSE) reported above: A717 is clearly and significantly inferior to the other methods, while DE is better than DASA and slightly better than PSO. In the other two scenarios (AO and NPO), there is no significant difference in performance between all four methods: PSO is handling the AO scenario slightly better than the other methods, while A717 performs better compared to the other methods (significantly better than DE) in the case of the NPO scenario when considering data with 5% noise.

**Figure 5:**
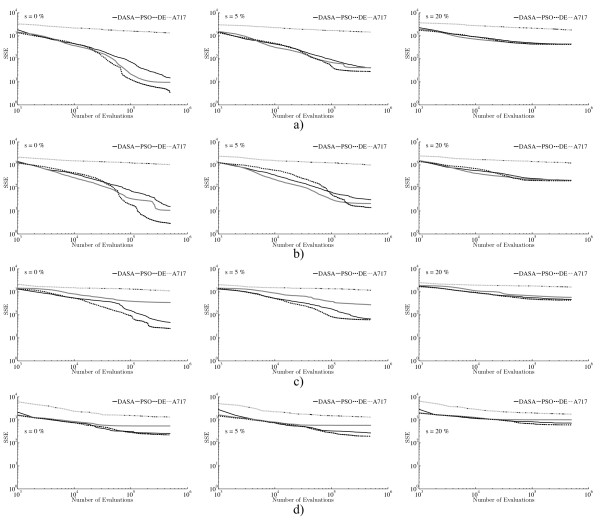
**Convergence performance of the optimization methods on the task of parameter estimation from artificial data**. Convergence curves of the four parameter estimation methods (DASA, PSO, DE, and A717) applied to three artificial datasets (columns) and four observation scenarios (rows): a) CO; b) AO; c) TO; and d) NPO. Graphs in the left column correspond to the noise-free data set, while the graphs in the middle and right column correspond to the noisy datasets with 5% and 20% relative noise, respectively. In order to capture the convergence trend over a wide range of values, the convergence curves are plotted using logarithmic scales for both axes.

The convergence curves in Figure 5 further confirm that DE is the most suitable method for parameter estimation in the endocytosis model for the given amount (half a million) of function evaluations. DE has faster convergence than DASA and PSO over all scenarios when considering noise-free data: in the CO and the TO case this is clear after 10 thousand evaluations, while in the AO and NPO case DE outperforms the others after one hundred thousand evaluations. The convergence rate of DE and the other methods is notably influenced by the noise level: regardless of the observation scenario, at 20% noise level there is no difference in the convergence rates of DASA, PSO, and DE, and it is clear that all methods (not only A717) have extremely slow convergence and seem to be trapped in local optima. Moreover, when comparing DASA and PSO, the convergence plots show that DASA is better in the TO and NPO scenario, while PSO has better convergence in the CO and AO scenario. A717 is clearly showing the poorest convergence, which is very little affected by the different observation and noise scenarios.

In order to assess the statistical significance of the differences in performance across all scenarios, two Holm tests were conducted using first the median values of RMSE and then the median values of RMSEm. The corresponding median values are given in Table 3, while the results of the Holm tests regarding both metrics are reported in Table 4. In terms of output reconstruction (RMSE), DE is the best ranked method that significantly outperforms the other three methods at the 0.05 significance level. In terms of complete system dynamics reconstruction (RMSEm), PSO is the best ranked method that significantly outperforms A717 at the same significance level, while the advantage over DASA and DE is not statistically significant. Finally, we check whether good output reconstruction is related to good overall system dynamics reconstruction, which is of central interest to the modeler. In Table 5, we check the validity of the conjecture that "best according to the output reconstruction is best according to the complete model reconstruction". For each model, simulated with the best parameter estimates obtained by a single method in a single case (a single observation scenario with a single data set), we report the corresponding RMSE and RMSEm values and expect that the optimization method that led to the best RMSE (the figure printed in bold in each row) would also lead to the best RMSEm (the figure printed in italic). This is trivially true in the CO scenario, where RMSE equals RMSEm, hence at all three noise levels DE leads to best RMSE and RMSEm. In the NPO scenario, this never happens, since DASA and PSO estimates lead to best RMSEm at different noise levels. In the other two scenarios, AO and TO, only at one out of three noise levels, DE leads to best RMSE and RMSEm; at the other two noise levels, the other meta-heuristic methods lead to best RMSEm. In sum, only in two out of nine non-trivial cases, models that perform best with respect to RMSE lead also to the best RMSEm performance.

**Table 3:** Results on RMSE and RMSEm of the models estimated from artificial data.

Noise	Scenario	RMSE				RMSEm			
			
		DASA	PSO	DE	A717	DASA	PSO	DE	A717
	CO	0.0651	0.0527	**0.0189**	0.7005	0.0651	0.0527	**0.0189**	0.7005
0%	AO	0.0625	0.0539	**0.0250**	0.6099	1.6272	**0.7866**	1.7876	1.0684
	TO	0.0951	0.1507	**0.0197**	0.6612	0.5857	0.4606	**0.2511**	0.7960
	NPO	0.2993	0.5040	**0.2282**	0.6881	2.6840	**2.0717**	3.9246	3.0273

	CO	0.1164	0.1121	**0.0999**	0.7287	0.1164	0.1121	**0.0999**	0.7287
5%	AO	0.0902	0.0861	**0.0690**	0.6232	1.0437	**0.9043**	1.4639	1.3442
	TO	0.1363	0.1341	**0.1006**	0.6546	0.6162	**0.2750**	0.2831	0.9265
	NPO	0.3162	0.5166	**0.2463**	0.6897	2.8668	3.8831	6.6315	**2.1172**

	CO	0.3958	0.3941	**0.3907**	0.8113	0.3958	0.3941	**0.3907**	0.8113
20%	AO	0.2770	0.2760	**0.2707**	0.6782	1.7547	**1.0050**	2.8513	1.3052
	TO	0.4023	0.3983	**0.3917**	0.7810	0.6967	0.4606	**0.4289**	0.9952
	NPO	0.4929	0.6407	**0.4585**	0.8023	**2.1250**	2.5423	2.8333	2.1999

The table presents the median values of RMSE and RMSEm (over the 25 runs) of the models reconstructed with the parameters' estimates obtained by the three optimization methods from artificial data. The best values for both metrics are given in bold.

**Table 4:** Results of the Holm test for significance level *α *= 0.05.

*i*	*α*/*i*	Method	RMSE			Method	RMSEm		
					
			*z_i_*	*p_i_*	Hypothesis		*z_i_*	*p_i_*	Hypothesis
3	0.017	A717	5.69	1.25·10^-8^	Rejected	A717	2.53	1.14·10^-2^	Rejected
2	0.025	DASA	3.16	1.57·10^-3^	Rejected	DASA	1.58	1.14·10^-1^	Accepted

1	0.050	PSO	2.53	1.14·10^-2^	Rejected	DE	1.58	1.14·10^-1^	Accepted

The table summarizes the outcome of the Holm test performed on the median values of the RMSE and RMSEm results obtained by parameter estimation with the four optimization methods from artificial data. In the first test based on median values of the RMSE measure, DE is the reference method with rank *i *= 0. In the second test based on median values of the RMSEm measure, PSO is the reference method with rank *i *= 0. The hypothesis "there is no difference in performance between DE (PSO) and the *i*-th ranked method" is rejected if the statement *p_i _*<*α*/*i *holds.

**Table 5:** The RMSE and RMSEm values for the best model estimated from artificial data.

Noise	Scenario	DASA		PSO		DE		A717	
					
		RMSE	RMSEm	RMSE	RMSEm	RMSE	RMSEm	RMSE	RMSEm
	CO	0.0345	0.0345	0.0430	0.0430	**0.0064**	*0.0064*	0.6080	0.6080
0%	AO	0.0446	6.0913	0.0406	*0.7693*	**0.0043**	1.1807	0.4644	21.3690
	TO	0.0468	1.0964	0.0447	*0.0877*	**0.0110**	0.1074	0.4542	0.6150
	NPO	0.2382	2.5430	0.3198	*1.9977*	**0.1774**	12.2287	0.6220	3.0273

	CO	0.1064	0.1064	0.1072	0.1072	**0.0977**	*0.0977*	0.5363	0.5362
5%	AO	0.0739	0.3343	0.0803	1.8387	**0.0678**	*0.2424*	0.3570	0.3723
	TO	0.1139	*0.1246*	0.1096	0.1639	**0.0985**	0.5058	0.4007	0.9028
	NPO	0.2562	3.0161	0.3349	*1.4970*	**0.2163**	318.415	0.5189	1.9670

	CO	0.3926	0.3926	0.3925	0.3925	**0.3904**	*0.3904*	0.6490	0.6490
20%	AO	0.2742	1.3904	0.2735	*0.4750*	**0.2704**	1.6916	0.4680	0.6220
	TO	0.3948	0.4568	0.3955	0.4368	**0.3913**	*0.3954*	0.5698	1.2933
	NPO	0.4616	*1.8011*	0.5055	2.6218	**0.4448**	5.4207	0.7556	7.2268

The table presents the RMSE and corresponding RMSEm values for the model simulated with the best parameters obtained by parameter estimation with the four optimization methods from artificial data. The best values for RMSE are marked in bold, while the best values for RMSEm are marked in italic.

### Parameter estimation with measured data

**Figure 6:**
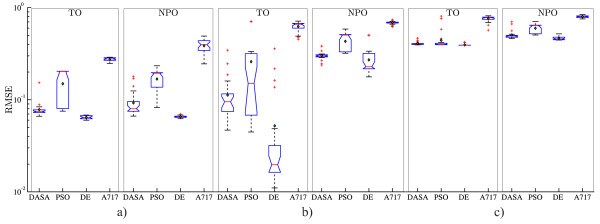
**RMSE performance of the models obtained by parameter estimation from measured data**. Boxplots of the performance distributions of the four optimization methods (DASA, PSO, DE, and A717) in terms of the reconstructed output (RMSE), when considering two different observability scenarios (columns TO and NPO) and three datasets: a) measured data, b) artificial data with *s *= 5% relative noise; and c) artificial data with *s *= 20% relative noise. Graphs b) and c) are the same as the corresponding graphs from Figure 3. Due to the large differences in the order of magnitude, the RMSEm values are plotted on a logarithmic scale.

Table 6 summarizes the results of parameter estimation in the Rab5-to-Rab7 conversion model using measurements obtained through real-world experiments. The rows Best, Median, and Worst give the RMSE value corresponding to the best, median and worst solution found by different optimization methods. The remaining two rows report the average RMSE performance (Average) and its standard deviation (Std). We consider only the observation scenarios TO and NPO, which are applicable given that the total protein concentrations are measured: the other two scenarios (CO and AO) are not applicable in this case. The first two graphs in Figure 6.a visually summarize these results. The remaining four graphs, corresponding to the artificial noisy data (omitting the noise-free data as less likely in practice), are given as a reference for comparison.

**Table 6:** Results on RMSE of the models estimated from measured data.

Scenario		DASA	PSO	DE	A717
	Best	0.0661	0.0752	**0.0599**	0.2482
	Median	0.0744	0.2032	**0.0643**	0.2782
TO	Worst	0.1530	0.2045	**0.0682**	0.2898
	Average	0.0782	0.1494	**0.0647**	0.2749
	Std	0.0163	0.0627	**0.0029**	0.0124

	Best	0.0665	0.0825	**0.0623**	0.2453
	Median	0.0799	0.1942	**0.0649**	0.3964
NPO	Worst	0.1788	0.2338	**0.0698**	0.4920
	Average	0.0924	0.1680	**0.0654**	0.3857
	Std	0.0305	0.0471	**0.0019**	0.0724

The table presents the RMSE values associated with the predicted models (over 25 runs) obtained by parameter estimation with the four optimization methods from measured data. The best values regrading all statistics are given in bold.

**Figure 7:**
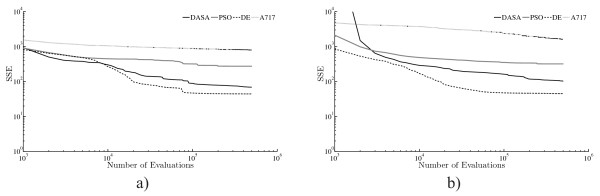
**Convergence performance of the optimization methods on the task of parameter estimation from measured data**. Convergence curves of the four parameter estimation methods (DASA, PSO, DE, and A717) when considering two observation scenarios: a) TO and b) NPO. In order to capture the convergence trend over a wide range of values the convergence curves are plotted using logarithmic scales for both axes.

The results on measured data confirm the findings of the experiments performed on artificial data. DE consistently leads to models with smallest RMSE (best performance), regardless of whether we consider the best, median, worst, or average RMSE (over the 25 runs). The boxplots clearly show the statistical significance of the performance differences between the four methods. DASA is the second best method, PSO is ranked third and A717 is ranked as the worst performing method. The observation about the higher variance of the RMSE values obtained by the PSO method in the TO and NPO scenarios with artificial data is confirmed in the experiments with measurement data. In the case of measured data, there is a very similar error distribution (range of values) in both scenarios (which is less expected given the definition of the scenarios), while in case the of artificial data, the NPO scenario is characterized with higher RMSE errors than the TO scenario. The error distribution in the measured data case is closer to the error distributions generated when considering artificial data with a noise level of 5%. Similarly, the convergence curves in Figure 7 resemble the ones for artificial data, i.e, the DE method converges faster to better solutions than the other three methods.

**Figure 8:**
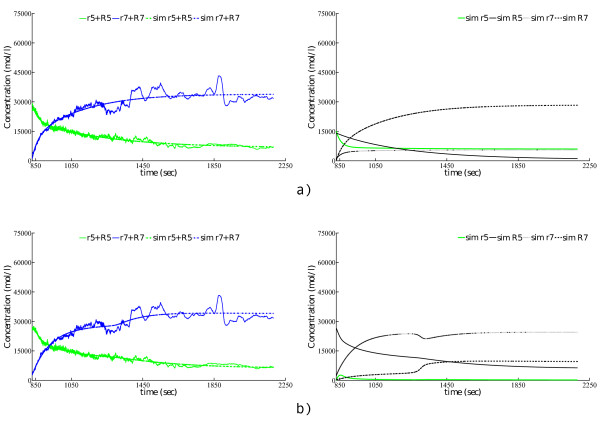
**Simulated behavior of the best models obtained by parameter estimation from measured data in the TO observation scenario**. Experimental (observed) vs. reconstructed output (left-hand side) and simulated behavior (right-hand side) of the model corresponding to the best parameters' values estimated from measured data in the TO observation scenario using: a) DASA and b) DE.

As a final test of the quality of the obtained models, we can visually compare the observed outputs with the outputs predicted by the models. In this context, Figure 8 visualizes the simulated output vs. the measured output (graphs on the left-hand side) and the complete dynamic behavior of all the system variables (graphs on the right-hand side) for the two models corresponding to the best parameters estimated by DASA and DE for the TO scenario. Additional file 1, Figures S1-S4 depict the predicted dynamics of the best model vs. the real-experimental behavior of the Rab5-to-Rab7 conversion for the TO and NPO scenarios found by DASA, PSO, DE, and A717, respectively. Since the time scale in the measured data was shifted for the sake of synchronization of the conversion events of Rab5 and Rab7 domain proteins, we rescaled it in order to conduct a direct comparison of the numeric simulation (reference model) with the measured data: We used the transformation *t *← 4*t *+ 850.

The left-hand side graphs in additional file 1, Figure S4 show that A717 fails to reconstruct the Rab5-to-Rab7 dynamics. In both observation scenarios, A717 fails to find a model with the cut-out switch behavior observed in Figure 2. On the other hand, DASA, PSO, and DE, when following the TO scenario, are able to almost perfectly reconstruct the real-experimental data (see left-hand side graph in: Figure 8.a, and additional file 1, Figures S2.a and S3.b), complete with the time point when the switch in the total (active- and passive-state) protein concentrations occurs. In the NPO scenario, DASA, PSO, and DE are also able to almost perfectly match the 'assumed' model output (active-state protein concentrations) against the measured system output (total protein concentration), but if we compare the model outputs, we can see that only DE is able to reconstruct the cut-out switch (even though slightly shifted on both scales, see graph on the left-hand side of additional file 1, Figure S3.b), while DASA and PSO are only able to fit one protein domain, due to the completely different dynamics of the simulated passive-state protein concentration (see graphs on the left-hand side of additional file 1, Figures S1.b and S2.b).

Finally, the analysis of reconstructed model dynamics (complete simulation of all the system variables) of the obtained models reveals further details about their quality. Note first that the simulated behavior in Figure 2 shows that the passive-state protein concentrations are almost constant all the time, leading to the conclusion that the cut-out switch appears at the very same time point for both total and active-state protein concentrations. For the TO scenario, only the model obtained with DASA has these two properties of the original model behavior (see its complete simulation on the right-hand side of Figure S3.a), while in the NPO scenario this holds for the model obtained by DE (see its complete simulation on the right-hand side of additional file 1, Figure S3.b). All the other models do not match one or both properties: the complete simulation graphs show that the concentrations of passive-state proteins vary and/or the switch of the active-state concentrations takes place at a different time point (or at all). Further comparison of the simulated behaviors with the original cut-out switch model behavior from Figure 2 also shows that the ratio between the active- and passive-state protein concentrations in the simulation of the model obtained with DASA in the TO case is closer to the same ratio in the original model.

Overall, the results on measured data show that all three meta-heuristic methods are far better than A717. Among the meta-heuristic methods, DE has a clear advantage over the other two methods, both in terms of the convergence rate and in terms of the reconstruction of model output. In terms of other relevant qualitative aspects of the behavior of the obtained models, i.e., the time point of the switch and the ratio between active- and passive-state protein concentrations, one of the other two methods (DASA) performs better then DE. However, note that these qualitative aspects have not been included in the objective function (SSE) used by the optimization methods: thus, we cannot objectively and fairly compare the methods along this dimension.

### Parameter values and practical parameter identifiability

Table 7 compares the reference parameter values from Eq. (11) with the best parameter values obtained using each of the four parameter estimation methods for the CO and TO scenarios on artificial data with 20% relative noise. Additional file 1, Tables S1-S4, present the same comparison in terms of relative error of the estimated parameters with respect to all four observation scenarios on artificial data with 0%, 5%, and 20% noise for DASA, PSO, DE, and A717, respectively, while additional file 1, Table S5 presents the best parameter values obtained by the four parameter estimation methods on measured data, for the TO and NPO observation scenarios. Despite the fact that DE finds parameter values that lead to low values of the objective function, the obtained parameter values differ quite substantially from the reference parameter values. Results on artificial data show this same pattern of large differences between the estimated and reference parameter values for all four parameter estimation methods in all scenarios; except for the parameters *c*_4_, *c*_8_, *c*_12_, *c*_15_, *r*_5_(0), and *r*_7_(0), the relative error of the other estimated parameters is over 100%. On measured data, we do not have reference values, but the comparison (additional file 1, Table S5) with the parameter values proposed by Del Conte-Zerial *et al*. [16] reveals the same pattern of large differences.

**Table 7:** Best estimated values of the model parameters obtained from artificial data with 20% noise.

		CO				TO			
			
*c*	c*	DASA	PSO	DE	A717	DASA	PSO	DE	A717
*c*_1_	1	4.0000	1.4644	0.2226	1.6393	3.1593	1.5627	1.8293	0.2974
*c*_2_	0.3	3.7099	2.0786	1.1132	2.5748	3.7499	2.7324	4	2.5086
*c*_3_	0.1	0.1977	1.2612	0.1974	0.0229	3.3526	0.2064	0.2682	0.5823
*c*_4_	2.5	3.5412	0.4192	3.1208	3.3226	0.2007	1.5837	3.7871	0.1709
*c*_5_	1	3.9940	1.4613	0.2217	1.5411	3.1287	1.4623	1.7688	0.5845
*c*_6_	0.483	0.5165	3.0640	3.6860	1.3897	0.4074	1.8952	0.4713	1.9140
*c*_7_	0.21	3.9471	2.6526	0.1503	1.5383	1.7030	2.9345	3.4951	2.0874
*c*_8_	3	3.1843	1.5314	3.4591	1.6254	1.5254	1.6742	3.1784	3.5895
*c*_9_	0.1	0.1563	1.5057	0.0524	3.1257	1.6444	1.5321	0.9762	1.9652
*c*_10_	0.021	2.0757	1.8316	0.0645	2.4551	0.2091	2.1490	1.4581	1.1780
*c*_11_	1	1.8340	2.9039	2.2013	2.3769	2.5222	3.2725	1.9195	2.6830
*c*_12_	3	3.1572	2.2358	1.7009	2.8349	1.2553	1.5096	0.1557	1.2227
*c*_13_	0.31	4.0000	1.7187	0.9381	0.6126	4.0000	2.9568	3.4364	3.2812
*c*_14_	0.3	1.0661	1.3179	0.3833	2.2955	1.7539	1.1975	0.8110	2.4085
*c*_15_	3	2.3525	1.7764	3.9800	3.6281	2.1599	2.1684	3.5535	3.3994
*c*_16_	0.483	0.5178	3.0728	3.6981	1.2091	0.4224	2.0041	0.7261	2.7693
*c*_17_	0.06	1.8635	0.4696	0.4159	2.0548	0.8316	1.3081	1.7687	0.2836
*c*_18_	0.15	2.7213	1.0043	0.1087	0.4984	0.5992	1.0744	1.3643	0.6677
*r*_5_(0)	1.0	0.8750	0.9116	0.9122	0.9535	0.9957	0.4830	0.9239	1.4011
*R*_5_(0)	0.001	4.0E-07	0.0358	0.1194	1.0854	3.3E-07	0.2313	0	1.3742
*r*_7_(0)	1.0	0.8096	1.3352	0.7978	0.1557	1.0153	0.4473	0.7310	1.0320
*R*_7_(0)	0.001	1.2E-10	0.2444	3.4E-04	0.8451	0.0139	0.1696	0.2515	0.9143

Best parameters' values as estimated by the four optimization methods from artificial data with 20% relative noise in the CO and TO observation scenarios.

Evidently, many quite different sets of parameter values produce behaviors that resemble the reference model behavior, suggesting that the endocytosis modeling task, as many others in system biology, has parameter identifiability problems. Indeed, a systematic study of seventeen system biology models [43] has found parameter identifiability issues in each of them. To empirically confirm this conjecture about our model, we performed a practical parameter identifiability test using the Monte Carlo-based approach and DE as a parameter estimation method. We considered this test in three observations scenarios, CO, AO, and TO, using data with 20% noise. The results of the test in the different observation scenarios confirm that the considered parameter estimation task has identifiability issues. The results reveal high relative errors; the mean value of the estimated parameters are overall far from the reference ones. Except for the parameters *c*_4_, *c*_8_, *c*_12_, *c*_14_, *c*_15_, *r*_5_(0), and *r*_7_(0), the relative error of the other estimated parameters is over 100%. This observation additionally re-confirms the statements in the previous paragraph obtained form the results regarding all optimization methods. In extreme cases, the relative errors are over 1000%, as it is the case with the parameters: *c*_10 _(in all scenarios), *c*_17 _(in CO and TO scenarios), *R*_5_(0) (in CO and TO scenarios), and *R*_7_(0) (in TO scenario). Furthermore, the calculated uncertainties (95% confidence interval) of the parameters are large, especially for *c*_7_, *R*_5_(0), and *R*_7_(0) over all scenarios; see additional file 1, Tables S6-S8 that summarize the results of the Monte Carlo-based approach for the CO, AO, and TO scenario, respectively.

**Figure 9:**
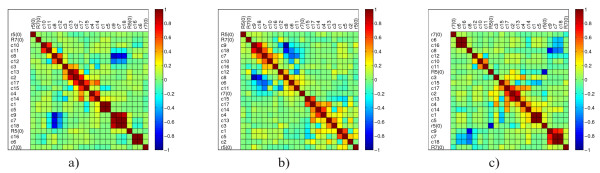
**Correlation matrices for the parameters' estimates obtained by DE from noisy data (***s *= 20**%) in a Monte Carlo-based approach**. Colored matrix cells visualize the correlation *R *for parameter pairs based on a scale-to-color mapping. The cells on the main diagonal represent the self-correlations of the parameters (they are equal to 1). The most correlated pairs of parameters per observations scenarios are: a) *R*(*c*_6_, *c*_16_) = 0.99997, *R*(*c*_1_, *c*_5_) = 0.99997, *R*(*c*_7_, *c*_18_) = 0.9862, *R*(*c*_7_, *c*_9_) = 0.9093, *R*(*c*_8_, *c*_9_) = -0.8749, *R*(*c*_9_, *c*_18_) = 0.8568, *R*(*c*_2_, *c*_13_) = 0.8413 in the case of CO; b) *R*(*c*_8_, *c*_9_) = -0.9097 and *R*(*c*_9_, *c*_18_) = -0.7789 in the case of AO; and c) *R*(*c*_6_, *c*_16_) = 0.9980, *R*(*c*_1_, *c*_5_) = 0.9934, *R*(*c*_7_, *c*_18_) = 0.9901, *R*(*r*_5_(0), *R*_5_(0) = -0.8509, *R*(*c*_2_, *c*_13_) = 0.8343, and *R*(*c*_8_, *c*_9_) = -0.8105 in the case of TO. Smallest values for R are obtained for the following pairs of parameters: a) *R*(*c*_1_, *c*_18_) = -0.00196 in the case of CO; b) *R*(*c*_3_, *c*_5_) = 0.000032 in the case of AO; and c) *R*(*c*_4_, *c*_6_) = -0.0011 in the case of TO.

**Figure 10:**
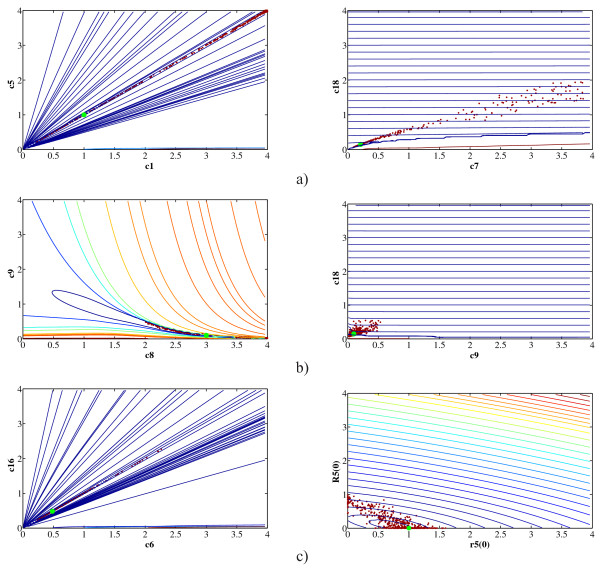
**Contour plots of the objective function with scatter plots of the parameters' estimates obtained by DE from noisy data (*s *= 20%) in a Monte Carlo-based approach**. The plots correspond to two representative pairs of correlated parameters in the observation scenarios: a) CO; b) AO; and c) TO. Note that one pair of correlated parameters in the TO observation scenario corresponds to the initial values of the Rab5 protein. The green dot represents the reference parameter value from Eqs. (11) and (12). The red dots are the parameters' estimates obtained by the DE method with the Monte Carlo-based approach.

Furthermore, the estimated values for many model parameters are evenly distributed across the parameter ranges; see the histograms of the distributions of the estimated parameter values in additional file 1, Figures S5-S7. If we take a look at the histograms for the CO scenario, we can see that parameters like *c*_1_, *c*_2_, *c*_5_, *c*_8_, *c*_11_, and *c*_13 _have very similar (almost) uniform distributions with higher concentration of the estimates on the bounds of the allowed range. We observe similar distributions for most of these parameters in the AO scenario (including *r*_5_(0), *r*_7_(0), *c*_6_, and *c*_16_) and in the TO scenario (including *c*_6_, *c*_15_, and *c*_16_) as well. For some parameters (like *c*_3_, *c*_12_, and *c*_13 _in the CO and AO scenario) the confidence interval does not include the reference value of the parameter, emphasizing the complexity of the optimization problem and the objective function. A closer look at the histograms reveals that some pairs of parameters have very similar (or almost identical) distributions of the estimates: This is in general the case with the (*c*_1_, *c*_5_) and (*c*_6_, *c*_16_) pairs of parameters. Note also how the distributions of the initial values of the system variables *r*_5_(0) and *r*_7_(0) differ among scenarios. In the case of complete observability, their values follow (almost) a Gaussian distribution around the reference value. In the TO scenario, most of the estimated initial values are in the neighborhood of the reference values even though the relative errors are higher than in the CO scenario. However, in the AO scenario, the distribution does not resemble a Gaussian; the values are spread all over the corresponding ranges, with higher concentrations at the ranges' limits and far from the reference values. Evidently, the lack of information on the concentration of passive-state proteins (*r*_5 _and *r*_7_) in the data worsens the problems related to parameter identifiability. The correlation matrices for the estimated parameter values, presented in Figure 9, re-confirm the practical identifiability problems, by emphasizing several pairs of correlated parameters. In the CO scenario, there are seven pairs of highly correlated parameters: A correlation *R *> 0.9 is evident for the (*c*_6_, *c*_16_), (*c*_1_, *c*_5_), (*c*_7_, *c*_18_), and (*c*_8_, *c*_9_) pairs of parameters, while the pairs (*c*_8_, *c*_9_), (*c*_8_, *c*_18_), and (*c*_2_, *c*_13_) have correlations in the range 0.84 < |*R*| < 0.9. In the AO scenario, the most correlated are *c*_8 _and *c*_9_, while in the TO scenario there are six such pairs: the pairs (*c*_6_, *c*_16_), (*c*_1_, *c*_5_), and (*c*_7_, *c*_18_) have almost prefect linear correlation *R *> 0.99, while the (*r*_5_(0), *R*_5_(0)), (*c*_2_, *c*_13_), and (*c*_8_, *c*_9_) pairs have a correlation in the range 0.81 < |*R*| < 0.85. In the last case, the correlation between the initial values of passive-state and active-state protein concentrations is expected, since we only observe their sum in the TO scenario. The high pairwise correlations can be observed visually in Figure 10, where the scatter plots of the obtained solutions are combined with the contour plots of the objective function landscape for selected pairs of parameters. For example, observe the long diagonal valley with many (almost) equally good solutions for the (*c*_1_, *c*_5_) and (*c*_6_, *c*_16_) pairs of parameters in Figure 10.a (left-hand side) and Figure 10.c (left-hand side). We observe a similar pattern of behavior for these pairs of parameters in both the CO and TO scenarios; see additional file 1, Figures S8 and S10. Figure 10.b, corresponding to the AO scenario, confirms that the objective function is characterized with elongated elliptical contours for the parameter pairs (*c*_8_, *c*_9_) as well. While the above-mentioned examples of correlated parameters are related to the lack of practical identifiability, the plot for the *c*_7 _and *c*_18 _parameters on the right-hand side in Figure 10.a (see additional file 1, Figure S10 for the TO case as well) indicates structural non-identifiability of *c*_7 _in the considered search interval; we observe a very large flat region in the part of the space 0.5 <*c*_18 _< 4, where *c*_7 _can take any value and does not influence the objective function. A similar observation holds for the *c*_9 _and *c*_18 _parameters in Figure 10.b (see additional file 1, Figure S8 for the CO case as well), in which case the *c*_9 _parameter seems to be structurally non-identifiable. Finally, the right-hand side plot in Figure 10.c re-confirms the correlation of the initial conditions: (*r*_5_(0) and *R*_5_(0)).

## Conclusions

In this paper, we address the task of parameter estimation in models of the dynamics of biological systems as considered in the field of system biology. In this context, it is typical that the considered models are nonlinear (due to the nonlinearity of the behavior of the modeled systems), have many parameters (are of high dimensionality), the measurements are imperfect (due to measurement noise), the system can be only partially observed (leading to incomplete or misinterpreted measurements). These properties make parameter estimation a challenging optimization problem, calling for the use of advanced optimization methods.

The focus of this paper is the use of meta-heuristic optimization methods for parameter estimation in dynamic system models typical of systems biology. We conduct an extensive experimental comparison of four optimization methods: the differential ant-stigmergy algorithm (DASA), particle-swarm optimization (PSO), and differential evolution (DE), all from the same class of meta-heuristic methods, as well as a local-search derivative-based method (A717). We compare these four methods as applied to a parameter estimation problem representative of the target class of problems described above. We use a a practically relevant model of endocytosis that captures the nonlinear dynamics of endosome maturation reflected in a cut-out switch transition between the Rab5 and Rab7 domain protein concentrations. The model is nonlinear and has many parameters. We compare the performance of the four optimization methods on this task along a number of dimensions, including the quality of reconstructing the observed system output (the measured quantities) and the complete model dynamics (all system variables, including unobserved ones), as well as the speed of convergence. Comparisons are made under different observation scenarios (full observability and different types of partial observability). We use both real (measured) data, containing partial observations of the system, and pseudo-experimental (artificial) data obtained by simulating the model and adding different amounts of artificial noise: The use of pseudo-experimental data allows us a more controlled study of the influence of noise and observability on the performance of the parameter estimation (optimization methods).

Noise in the measurements does influence the performance of the optimization methods, with higher amounts of noise making the task more difficult. The observability of the system (as varied through the observation scenarios), has a much stronger influence, where less complete observations make the optimization task much more difficult. Worst results are obtained when the observations are misinterpreted, i.e., when the actual total concentrations of Rab5 and Rab7 are taken to represent the concentrations of these proteins in their active states.

We also investigate the practical identifiability of the model parameters: Like many similar tasks in systems biology, the task considered has parameter identifiability problems. These are manifested by high relative errors of the reconstructed parameter values, spread uniform-like distributions of some parameter estimates, and strong correlations between some pairs of estimated parameters. The problems are present in all observation scenarios and are most severe in the case of incomplete observations. The performance of all three meta-heuristic methods is affected by these problems. On the other hand, this explains the severe difficulties that the local search method (A717) experienced on the given parameter estimation task. Overall, the global meta-heuristic methods (DASA, PSO, and DE) clearly and significantly outperform the local derivative-based method (A717). Among the three meta-heuristics, differential evolution (DE) performs best in terms of the objective function, i.e., the quality of reconstructing the expected output, and in terms of the speed of convergence. These results hold for both real and artificial data, for all observability scenarios considered, and for all amounts of noise added to the artificial data. In terms of the quality of reconstructing the complete model dynamics and other qualitative aspects of the behavior of the obtained models, the different meta-heuristic methods exhibit different behavior and relative performance under different conditions: More work needs to be done to better understand and objectively evaluate these differences in performance.

Further work is needed to confirm and strengthen the conclusions drawn from the experimental evaluation presented in this paper, primarily in the direction of conducting additional experiments. On one hand, we need to test the optimization methods on other tasks of parameter estimation in nonlinear models of biochemical kinetics. On the other hand, we can extend the set of optimization methods applied to the parameter estimation tasks, considering other state-of-the-art algorithms used for parameter estimation in the domain of computational systems biology [7-9].

Last, but not least, we need to formalize relevant qualitative aspects of model quality (such as the time point of switch between the observed Rab5 and Rab7 concentrations in the endocytosis model) and include these in the formulation of the optimization problem of parameter estimation. These aspects will typically depend on domain knowledge about the particular problem at hand and can be made a part of the overall objective function or formulated as a separate objective function in a multi-objective optimization setting. This will allow us to objectively and fairly evaluate and compare the different optimization approaches from these aspects.

In sum, the bio-inspired meta-heuristic optimization methods considered are suitable for estimating the parameters in the ODE model of the dynamics of endocytosis under a range of conditions. The model considered, as well as the observational conditions (such as partial observability and noise) are representative of parameter estimation tasks in ODE models of biochemical network dynamics. Thus, our results point out and clearly highlight the promise of bio-inspired meta-heuristic methods for solving problems of parameter estimation in models of dynamic systems from the area of system biology.

## Authors' contributions

SD and JŠ initiated the work, PK and KT implemented and adapted the optimization algorithms. KT performed the experimental evaluation of the algorithms and drafted the manuscript. LT, SD, and JŠ gave KT valuable advice on a variety of issues related to the manuscript revisions. All authors read and approved the final manuscript.

## Supplementary Material

Additional file 1 **Supplemental information**. This file contains Figures S1-S10 and Tables S1-S8 with results obtained from parameter estimation in the Rab-to-Rab7 conversion model. Experimental behavior vs. simulated behavior of the reconstructed output and reconstructed model dynamics with the best parameters estimated by DASA (Figure S1), PSO (Figure S2), DE (Figure S3), and A717 (Figure S4) using measured data. Relative errors of the best estimated parameters by DASA (Table S1), PSO (Table S2), DE (Table S3), and A717 (Table S4) using artificial data. Parameter values associated with the best solutions estimated using measured data (Table S5). Summary of results on the DE estimated parameters with the Monte Carlo-based approach using data with 20% noise in three observation scenarios: CO (Table S6), AO (Table S7), and TO (Table S8). Corresponding histograms of the DE estimated parameters with the Monte Carlo-based approach using data with 20% noise: CO (Figure S5), AO (Figure S6), and TO (Figure S7). Scatter plots of the Monte Carlo-based DE parameter estimates combined with contour plots of the objective function when considering data with 20% noise for the most correlated pairs of parameters in the CO (Figure S8), AO (Figure S9), and TO (Figure S10) observation scenarios. Click here for file
