# Supplementary material for: Parameter estimation with bio-inspired meta-heuristic optimization: modeling the dynamics of endocytosis
Source: BMC Syst Biol. 2011 Oct 11;5:159. doi: 10.1186/1752-0509-5-159 (PMC3271279; doi:10.1186/1752-0509-5-159)
Supplement: Additional file 1 — Supplemental information. This file contains Figures S1-S10 and Tables S1-S8 with results obtained from parameter estimation in the Rab-to-Rab7 conversion model. Experimental behavior vs. simulated behavior of the reconstructed output and reconstructed model dynamics with the best parameters estimated by DASA (Figure S1), PSO (Figure S2), DE (Figure S3), and A717 (Figure S4) using measured data. Relative errors of the best estimated parameters by DASA (Table S1), PSO (Table S2), DE (Table S3), and A717 (Table S4) using artificial data. Parameter values associated with the best solutions estimated using measured data (Table S5). Summary of results on the DE estimated parameters with the Monte Carlo-based approach using data with 20% noise in three observation scenarios: CO (Table S6), AO (Table S7), and TO (Table S8). Corresponding histograms of the DE estimated parameters with the Monte Carlo-based approach using data with 20% noise: CO (Figure S5), AO (Figure S6), and TO (Figure S7). Scatter plots of the Monte Carlo-based DE parameter estimates combined with contour plots of the objective function when considering data with 20% noise for the most correlated pairs of parameters in the CO (Figure S8), AO (Figure S9), and TO (Figure S10) observation scenarios. [file 1752-0509-5-159-S1.PDF]

Additional file

Parameter estimation with bio-inspired meta-heuristic optimization: modeling dynamics of endocytosis

Katerina Tashkova*[1], Peter Korošec[1], Jurij Šilc[1], Ljupčo Todorovski[2], Sašo Džeroski[3]

[1]Computer Systems Department, Jožef Stefan Institute, Jamova cesta 39, SI-1000 Ljubljana, Slovenia
[2]Faculty of Administration, University of Ljubljana, Gosarjeva ulica 5, SI-1000 Ljubljana, Slovenia
[3]Department of Knowledge Technologies, Jožef Stefan Institute, Jamova cesta 39, SI-1000 Ljubljana, Slovenia

Email: Katerina Tashkova*- katerina.taskova@ijs.sik; Peter Korošec - peter.korosec@ijs.si; Jurij Šilc - jurij.silc@ijs.si; Ljupčo Todorovski - ljupco.todorovski@fu.uni-lj.si; Sašo Džeroski - saso.dzeroski@ijs.si;

*Corresponding author

List of Figures

Figure S1. Simulated behavior of the best models obtained by DASA on measured data

Figure S2. Simulated behavior of the best models obtained by PSO on measured data

Figure S3. Simulated behavior of the best models obtained by DE on measured data

Figure S4. Simulated behavior of the best models obtained by A717 on measured data

Figure S5. Histograms of the parameters' estimates by DE on noisy data ($s = 20\%$) regarding the CO observation scenario in a Monte Carlo-based approach

Figure S6. Histograms of the parameters' estimates by DE on noisy data ($s = 20\%$) regarding the AO observation scenario in a Monte Carlo-based approach

Figure S7. Histograms of the parameters' estimates by DE on noisy data ($s = 20\%$) regarding the TO observation scenario in a Monte Carlo-based approach

Figure S8. Contour plots of the objective function with scatter plots of the parameters' estimates obtained by DE on noisy data ($s = 20\%$) regarding the CO observation scenario in a Monte Carlo-based approach

Figure S9. Contour plots of the objective function with scatter plots of the parameters' estimates obtained by DE on noisy data ($s = 20\%$) regarding the AO observation scenario in a Monte Carlo-based approach

Figure S10. Contour plots of the objective function with scatter plots of the parameters' estimates obtained by DE on noisy data ($s = 20\%$) regarding the TO observation scenario in a Monte Carlo-based approach

List of Tables

Table S1. Relative errors of the best parameter values estimated by DASA on artificial data

Table S2. Relative errors of the best parameter values estimated by PSO on artificial data

Table S3. Relative errors of the best parameter values estimated by DE on artificial data

Table S4. Relative errors of the best parameter values estimated by A717 on artificial data

Table S5. Best parameter values estimated by the four optimization methods on measured data.

Table S6. Summary of statistics over the estimated parameters by DE on noisy data ($s = 20\%$) regarding the CO observation scenario in a Monte Carlo-based approach

Table S7. Summary of statistics over the estimated parameters by DE on noisy data ($s = 20\%$) regarding the AO observation scenario in a Monte Carlo-based approach

Table S8. Summary of statistics over the estimated parameters by DE on noisy data ($s = 20\%$) regarding the TO observation scenario in a Monte Carlo-based approach

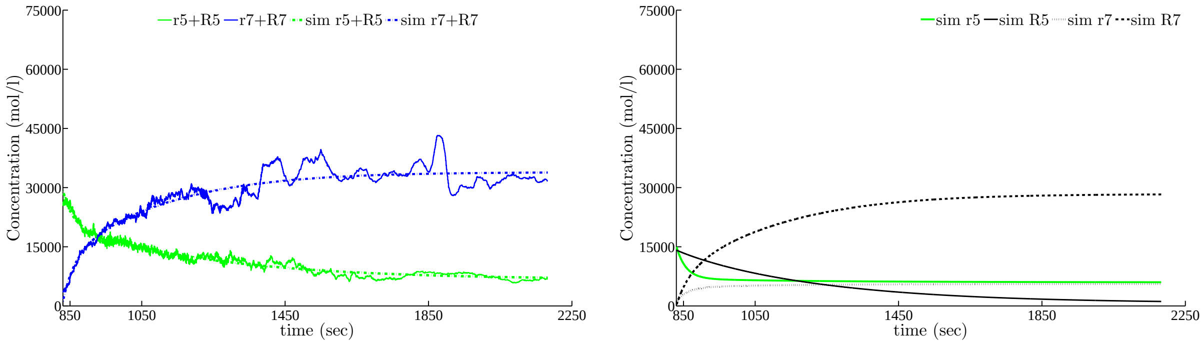

a)

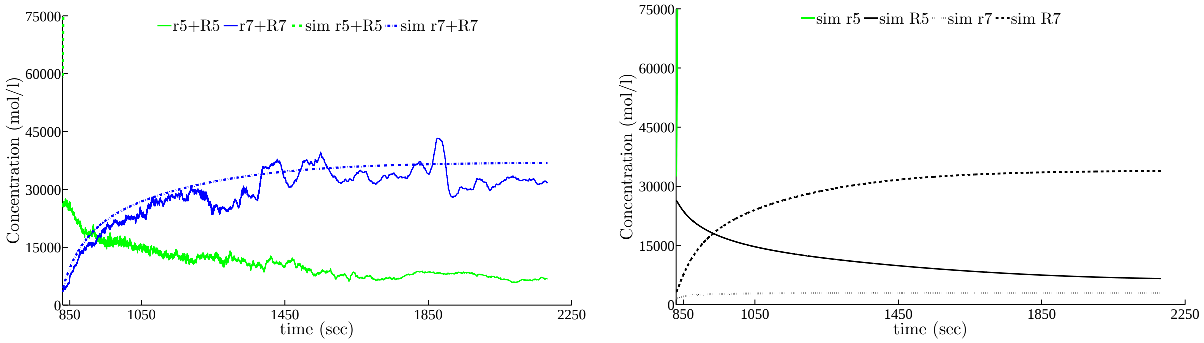

b)

Figure 1: **Simulated behavior of the best models obtained by DASA on measured data.** Experimental (observed) behavior vs. predicted (simulated) behavior shown in terms of the reconstructed output (left-hand side) and the reconstructed model dynamics (right-hand side) of the model with the best parameters estimated by DASA on measured data in the observation scenarios: a) TO and b) NPO. Note that in the case of NPO scenario, the simulated concentration of the passive-state Rab5 protein ("sim r5", green solid line) is instantly and rapidly increasing towards higher values until it reaches some stable level (approx. 200000 mol/l), therefore it is invisible on the right-hand graph. Related to this, the predicted total Rab5 concentration ("sim r5+R5", green dashed-dotted line) is almost invisible on the left-hand graph as well.

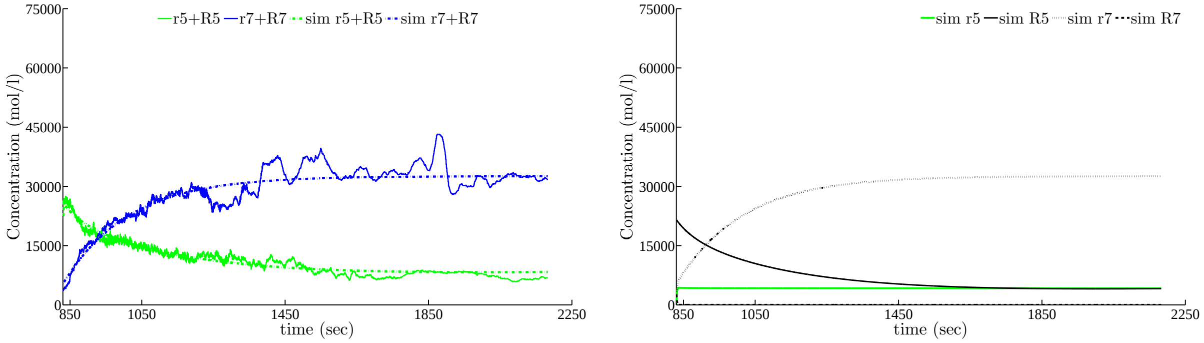

a)

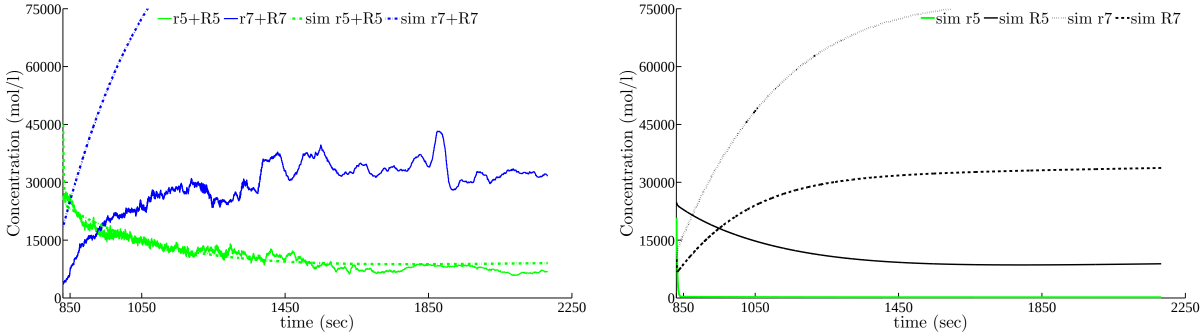

b)

Figure 2: **Simulated behavior of the best models obtained by PSO on measured data.** Experimental behavior vs. simulated behavior of the reconstructed output (left-hand side) and reconstructed model dynamic (right-hand side) with the best parameters estimated by PSO on measured data in the observation scenarios: a) TO and b) NPO. Note that in the case of TO scenario, the simulated concentration of the active-state Rab7 protein ("sim R7", black dashed line) is instantly and rapidly decreasing towards zero, therefore it is invisible on the right-hand graph. Similar behavior is observed in the NPO scenario, but in this case the simulated concentration of the passive-state Rab5 protein ("sim r5", green solid line) is instantly and rapidly decreasing towards zero, therefore it is invisible on the right-hand graph.

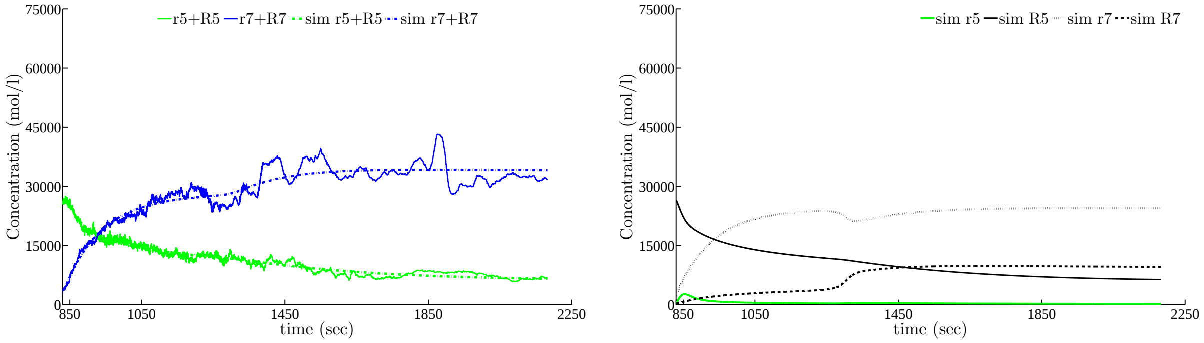

a)

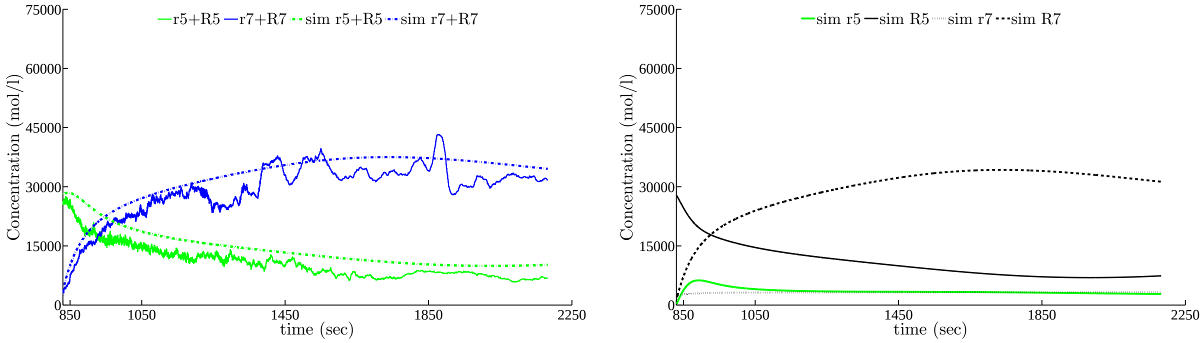

b)

Figure 3: **Simulated behavior of the best models obtained by DE on measured data.** Experimental (observed) behavior vs. predicted (simulated) behavior shown in terms of the reconstructed output (left-hand side) and the reconstructed model dynamics (right-hand side) of the model with the best parameters estimated by DE on measured data in the observation scenarios: a) TO and b) NPO.

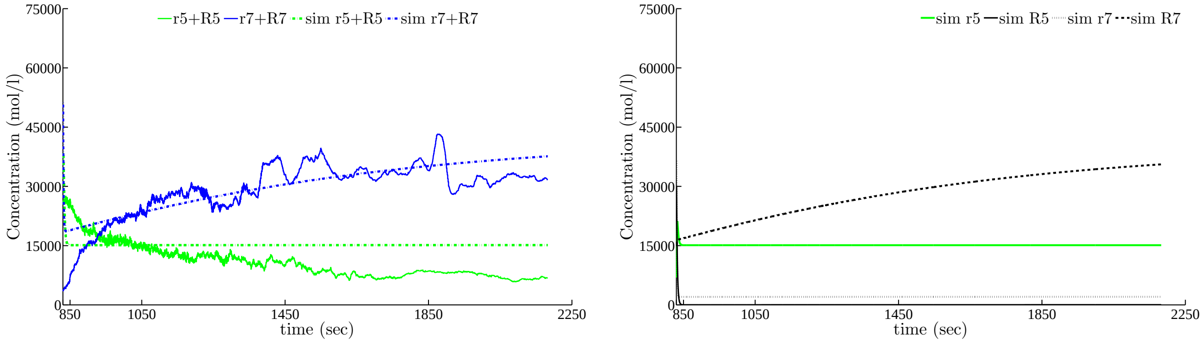

a)

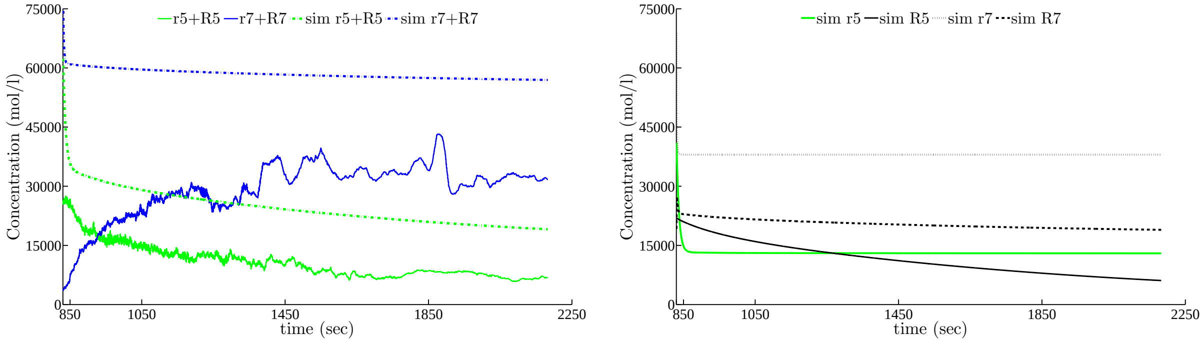

b)

Figure 4: **Simulated behavior of the best models obtained by A717 on measured data.** Experimental (observed) behavior vs. predicted (simulated) behavior shown in terms of the reconstructed output (left-hand side) and the reconstructed model dynamics (right-hand side) of the model with the best parameters estimated by A717 on measured data in the observation scenarios: a) TO and b) NPO. Note that in the case of TO scenario, the simulated concentration of the active-state Rab5 protein ("sim R5", black solid line) is instantly and rapidly decreasing towards zero, therefore it is almost invisible on the right-hand graph.

Table 1: **Relative errors of the best parameter values estimated by DASA on artificial data.** The relative errors of the estimated parameters c are calculated with regard to the reference values c^* according to the formula $\frac{|c^*-c|}{c^*}$. Note that the error values are given in percent [%].

		CO			AO			TO			NPO		
c	c^*	$s=0\%$	$s=5\%$	$s=20\%$	$s=0\%$	$s=5\%$	$s=20\%$	$s=0\%$	$s=5\%$	$s=20\%$	$s=0\%$	$s=5\%$	$s=20\%$
c_1	1	300	216	17	220	289	177	60	250	31	300	300	300
c_2	0.3	435	1150	803	691	479	1150	482	1176	658	1173	1199	1137
c_3	0.1	922	3253	156	100	100	100	3900	3890	13	224	88	98
c_4	2.5	91	92	6	59	60	57	91	89	24	18	12	42
c_5	1	281	213	3	31	4	42	94	300	70	300	300	299
c_6	0.483	9	16	432	433	621	683	60	43	499	13	46	7
c_7	0.21	1798	711	1799	205	1466	1776	1805	1207	816	672	560	1780
c_8	3	31	49	26	10	9	19	30	36	13	18	17	6
c_9	0.1	3891	1544	605	3899	3900	3900	31	562	8	254	307	56
c_{10}	0.021	12828	896	7765	1472	7928	10212	5531	5635	1679	1527	9160	9784
c_{11}	1	73	152	292	119	153	104	49	150	37	52	228	83
c_{12}	3	98	58	82	98	99	96	33	34	29	26	51	5
c_{13}	0.31	307	1190	651	813	1094	1179	1190	997	1175	1018	1190	1190
c_{14}	0.3	548	485	171	181	156	156	172	563	51	58	298	255
c_{15}	3	3	28	33	33	33	33	31	65	18	33	24	22
c_{16}	0.483	396	13	474	102	683	353	619	56	728	13	46	7
c_{17}	0.06	759	1286	2886	795	28	296	4042	308	3158	1653	3728	3006
c_{18}	0.15	220	299	1275	413	811	2055	327	1178	556	579	481	1714
$r_5(0)$	1	2	0.4	29	59	60	49	100	67	97	6	3	13
$R_5(0)$	0.001	19	100	203	139349	156945	159590	92	100	402	96	51	100
$r_7(0)$	1	96	2	93	59	50	93	65	93	91	1	1	19
$R_7(0)$	0.001	85883	1292	36882	50110	23812	48395	80	521	985	54	95	100

Table 2: **Relative errors of the best parameter values estimated by PSO on artificial data.** The relative errors of the estimated parameters c are calculated with regard to the reference values c^* according to the formula $\frac{|c^*-c|}{c^*}$. Note that the error values are given in percent.

		CO			AO			TO			NPO		
c	c^*	$s=0\%$	$s=5\%$	$s=20\%$	$s=0\%$	$s=5\%$	$s=20\%$	$s=0\%$	$s=5\%$	$s=20\%$	$s=0\%$	$s=5\%$	$s=20\%$
c_1	1	221	83	300	100	100	300	54	57	75	25	22	78
c_2	0.3	272	1233	600	1115	68	260	1233	786	398	1233	873	271
c_3	0.1	90	168	168	97	3754	100	119	117	107	116	134	97
c_4	2.5	9	51	38	70	100	60	28	31	7	37	27	25
c_5	1	246	77	296	100	100	140	38	60	90	25	22	78
c_6	0.483	417	2	142	725	720	191	728	250	251	10	19	663
c_7	0.21	8	1564	1078	99	100	97	72	49	45	19	44	28
c_8	3	11	6	44	33	33	12	19	33	17	14	5	15
c_9	0.1	37	876	1084	1944	1363	1574	58	75	52	43	37	48
c_{10}	0.021	258	6843	970	104	100	100	404	3166	2252	250	195	207
c_{11}	1	140	92	127	300	100	278	269	291	226	118	66	120
c_{12}	3	42	95	80	29	33	21	57	45	49	43	37	43
c_{13}	0.31	182	1009	776	715	86	54	1139	796	1188	1111	1151	203
c_{14}	0.3	28	170	12	501	508	493	20	41	16	66	19	28
c_{15}	3	5	18	43	33	33	33	36	13	10	10	42	33
c_{16}	0.483	408	50	153	37	98	53	285	190	559	11	19	666
c_{17}	0.06	438	2848	94	475	99	894	17	1272	909	2243	100	593
c_{18}	0.15	7	810	593	93	95	89	37	36	21	17	41	28
$r_5(0)$	1	10	8	12	58	6	23	99	92	100	0.2	4	9
$R_5(0)$	0.001	11550	100	86	79314	123582	147319	333	59	99	47	3807	11839
$r_7(0)$	1	0.5	27	24	55	80	32	59	100	10	0.1	3	20
$R_7(0)$	0.001	57	25050	50	113333	157207	107106	100	100	100	73	51	66

Table 3: **Relative errors of the best parameter values estimated by DE on artificial data.** The relative errors of the estimated parameters c are calculated with regard to the reference values c^* according to the formula $\frac{|c^*-c|}{c^*}$. Note that the error values are given in percent.

		CO			AO			TO			NPO		
c	c^*	$s = 0\%$	$s = 5\%$	$s = 20\%$	$s = 0\%$	$s = 5\%$	$s = 20\%$	$s = 0\%$	$s = 5\%$	$s = 20\%$	$s = 0\%$	$s = 5\%$	$s = 20\%$
c_1	1	115	56	208	140	115	255	158	211	90	209	264	46
c_2	0.3	816	811	919	797	1082	774	763	562	570	740	1174	593
c_3	0.1	59	106	242	45	71	83	185	1246	132	1043	111	1161
c_4	2.5	4	37	4	35	19	42	42	78	15	81	1	83
c_5	1	124	46	173	61	110	56	51	16	143	210	264	46
c_6	0.483	256	292	501	649	398	590	237	440	441	520	553	534
c_7	0.21	1226	1297	1107	730	1585	895	1349	1251	1025	328	694	1163
c_8	3	55	44	28	6	8	7	49	53	42	44	42	49
c_9	0.1	1943	1432	585	3654	3642	3439	1395	1808	912	834	834	1406
c_{10}	0.021	12792	10133	14304	11686	17748	17352	10473	3563	14414	11948	17065	8622
c_{11}	1	188	227	136	179	200	188	157	165	156	186	161	190
c_{12}	3	3	50	15	30	23	29	3	26	0.4	3	13	25
c_{13}	0.31	757	854	592	551	541	875	1025	697	506	362	727	454
c_{14}	0.3	212	299	150	208	161	218	179	168	237	201	126	339
c_{15}	3	35	28	19	28	12	17	11	18	46	25	12	41
c_{16}	0.483	245	315	521	227	204	150	161	211	372	521	555	536
c_{17}	0.06	1709	2080	3018	42	11	429	3629	1985	844	908	3044	683
c_{18}	0.15	500	616	784	813	1298	1241	898	983	734	183	425	570
$r_5(0)$	1	3	52	56	91	65	48	26	14	98	7	32	9
$R_5(0)$	0.001	96663	23031	21794	21315	68210	79688	53029	45843	13858	37554	82844	3481
$r_7(0)$	1	84	55	57	53	70	23	26	44	74	8	29	34
$R_7(0)$	0.001	38304	16858	54415	46707	23580	5703	23531	50995	55932	18971	21861	24338

Table 4: **Relative errors of the best parameter values estimated by A717 on artificial data.** The relative errors of the estimated parameters c are calculated with regard to the reference values c^* according to the formula $\frac{|c^*-c|}{c^*}$. Note that the error values are given in percent.

		CO			AO			TO			NPO		
c	c^*	$s=0\%$	$s=5\%$	$s=20\%$	$s=0\%$	$s=5\%$	$s=20\%$	$s=0\%$	$s=5\%$	$s=20\%$	$s=0\%$	$s=5\%$	$s=20\%$
c_1	1	200	70	166	281	260	176	31	264	165	220	228	64
c_2	0.3	301	736	906	738	768	866	765	892	862	809	1226	758
c_3	0.1	1327	482	295	100	503	2284	1408	383	2566	334	165	77
c_4	2.5	98	93	84	38	66	99	75	6	86	66	43	33
c_5	1	136	42	284	6	87	63	82	296	256	196	237	54
c_6	0.483	136	296	29	705	397	525	645	676	164	456	403	188
c_7	0.21	1231	894	611	6	1253	1213	22	1597	1369	226	1112	633
c_8	3	18	20	44	80	29	9	40	20	13	6	15	46
c_9	0.1	1183	1865	2528	2815	2792	2588	574	741	10	2193	443	3026
c_{10}	0.021	13058	5510	5534	9537	12926	11291	11765	15748	10253	14053	16837	11591
c_{11}	1	6	168	155	221	155	209	199	75	77	53	272	138
c_{12}	3	12	59	1	36	23	40	18	11	8	15	75	6
c_{13}	0.31	163	958	750	963	435	1000	653	962	121	583	157	98
c_{14}	0.3	735	703	1069	171	31	19	464	784	55	744	263	665
c_{15}	3	82	13	71	33	10	2	11	80	22	22	75	21
c_{16}	0.483	170	473	500	453	126	69	67	677	130	469	599	150
c_{17}	0.06	872	373	692	1368	251	3186	376	1306	649	2784	3636	3325
c_{18}	0.15	1027	345	67	47	1743	2312	1367	1379	2060	290	848	232
$r_5(0)$	1	51	40	7	0.1	98	23	72	34	26	45	8	5
$R_5(0)$	0.001	18553	137320	131935	31923	57318	39264	63014	141434	29665	38180	113730	108441
$r_7(0)$	1	68	3	17	56	49	26	13	60	92	20	67	84
$R_7(0)$	0.001	58815	91328	90558	52936	154684	83854	84335	142195	67290	125217	136966	84406

Table 5: **Best parameter values estimated by the four optimization methods on measured data.** Note that the initial values of the protein concentrations are scaled to simplify the comparison with the reference solution c^* in the artificial case.

		DASA		PSO		DE		A717	
c	c^*	TO	NPO	TO	NPO	TO	NPO	TO	NPO
c_1	1	0.0430	3.9990	0.7322	0.0169	0.0024	0.0124	2.3811	0.3542
c_2	0.3	3.9933	3.9998	2.2210	2.2310	3.9942	0.7024	2.4955	1.4670
c_3	0.1	2.0998	2.6714	1.3881	0.1878	0.0285	0.5391	3.6488	2.1391
c_4	2.5	3.8081	3.3911	3.7274	0.4415	1.4343	4.0000	1.8106	3.3485
c_5	1	0.1554	0.5537	3.7698	1.9905	0.2493	0.0908	3.4111	0.5914
c_6	0.483	0.0525	0.0717	0.0180	0.0425	0.0268	0.1561	0.1594	3.6601
c_7	0.21	3.8982	0.9565	1.3462	0.0848	0.1528	1.1262	1.1800	0.3755
c_8	3	2.4040	4.0000	1.4918	1.5146	3.9626	3.7725	0.6442	0.4293
c_9	0.1	0.4421	0.4149	1.6716	2.1492	0.0004	0.6144	3.9632	3.1055
c_{10}	0.021	2.4798	1.7236	2.8433	1.8045	0.0676	0.7280	3.0716	3.6792
c_{11}	1	0.0665	1.7365	2.7186	0.0003	0.4900	0.2363	2.9422	0.4156
c_{12}	3	0.0082	0.0149	3.7046	2.5726	3.9985	1.7607	3.6026	0.6001
c_{13}	0.31	0.0401	0.7091	3.7703	0.9808	1.7516	0.2506	1.2693	1.6567
c_{14}	0.3	2.9980	3.0230	2.8314	3.1975	1.1150	1.3669	3.5432	3.8190
c_{15}	3	3.5816	2.1751	2.1126	1.9293	3.8053	2.5061	1.5805	3.4274
c_{16}	0.483	0.3749	0.9534	0.0219	0.0216	0.0435	1.9111	3.0877	3.8228
c_{17}	0.06	0.0106	0.0184	0.0097	0.0075	0.0053	0.0137	1.7533	0.0078
c_{18}	0.15	0.6259	0.1223	3.0210	3.1291	0.4060	0.0895	0.0122	3.6839
$r_5(0)$	1	0.6917	1.5009	0.0231	0.9616	0.0100	0.0100	0.3196	1.8947
$R_5(0)$	0.001	0.6532	1.2222	0.9929	1.1451	1.2256	1.2863	1.3786	1.0128
$r_7(0)$	1	0.0212	0.0154	0.0688	0.2109	0.0611	0.0100	0.9175	1.7340
$R_7(0)$	0.001	0.0100	0.0835	0.0662	0.2533	0.0113	0.0515	0.3739	0.4843

Table 6: **Summary of statistics over the estimated parameters by DE on noisy data ($s = 20\%$) regarding the CO observation scenario in a Monte Carlo-based approach**. The column μ represents the mean values of estimated parameters; σ represents the standard deviation of the estimated parameters; $\frac{|c^*-\mu|}{c^*}$ is the relative error; c^l is the 2.5 percentile of the sample values, the lower bound of the 95% confidence interval; c^m is the 50 percentile of the sample values, the median; c^u is the 97.5 percentile of the sample values, the upper bound of the 95% confidence interval; $CI = c^u - c^l$ is the length of the 95% confidence interval; $\frac{CI}{\mu}$ is the confidence interval with respect to the mean; the last column represents the number of outliers. The complete sample size is 1000. For calculating the statistics we used reduced sample with size 428, without outliers.

c	c^*	μ	σ	$\frac{\|c^*-\mu\|}{c^*}$ [%]	c^l	c^m	c^u	CI	$\frac{CI}{\mu}$ [%]	outliers
c_1	1	2.529	1.419	152.86	0.377	3.143	3.998	3.621	143.21	38
c_2	0.3	2.802	1.027	834.12	0.556	3.023	4.000	3.444	122.91	0
c_3	0.1	0.212	0.024	111.62	0.160	0.214	0.257	0.096	45.52	140
c_4	2.5	3.327	0.427	33.07	2.369	3.359	3.999	1.630	49.01	96
c_5	1	2.529	1.419	152.91	0.378	3.154	4.000	3.622	143.20	0
c_6	0.483	0.582	0.322	20.49	0.265	0.487	1.620	1.356	232.95	206
c_7	0.21	0.832	1.109	296.43	0.117	0.233	3.643	3.526	423.58	65
c_8	3	2.907	0.856	3.08	1.512	3.003	4.000	2.487	85.54	0
c_9	0.1	0.306	0.381	206.29	0.024	0.110	1.361	1.337	436.62	55
c_{10}	0.021	0.397	0.393	1788.55	0.032	0.247	1.497	1.465	369.39	98
c_{11}	1	2.680	0.795	167.99	1.363	2.683	3.998	2.636	98.36	0
c_{12}	3	1.630	0.388	45.66	0.922	1.616	2.491	1.569	96.24	40
c_{13}	0.31	3.002	1.009	868.38	0.571	3.312	4.000	3.429	114.23	0
c_{14}	0.3	0.476	0.162	58.60	0.165	0.495	0.764	0.599	125.90	52
c_{15}	3	2.386	0.713	20.45	1.250	2.298	3.991	2.741	114.86	0
c_{16}	0.483	0.582	0.322	20.49	0.264	0.488	1.626	1.362	233.99	194
c_{17}	0.06	0.746	0.612	1142.63	2.99E-04	0.638	1.951	1.951	261.67	4
c_{18}	0.15	0.443	0.515	195.34	0.087	0.168	1.776	1.689	381.30	26
$r_5(0)$	1	0.980	0.193	1.95	0.626	0.976	1.407	0.782	79.73	31
$R_5(0)$	0.001	0.013	0.019	1210.78	0	0.003	0.069	0.069	524.15	107
$r_7(0)$	1	0.992	0.140	0.79	0.695	0.993	1.262	0.567	57.16	55
$R_7(0)$	0.001	0.003	0.005	171.37	0	3.90E-04	0.018	0.018	665.52	161

Table 7: **Summary of statistics over the estimated parameters by DE on noisy data ($s = 20\%$) regarding the AO observation scenario in a Monte Carlo-based approach**. The column μ represents the mean values of estimated parameters; σ represents the standard deviation of the estimated parameters; $\frac{|c^*-\mu|}{c^*}$ is the relative error; c^l is the 2.5 percentile of the sample values, the lower bound of the 95% confidence interval; c^m is the 50 percentile of the sample values, the median; c^u is the 97.5 percentile of the sample values, the upper bound of the 95% confidence interval; $CI = c^u - c^l$ is the length of the 95% confidence interval; $\frac{CI}{\mu}$ is the confidence interval with respect to the mean; the last column represents the number of outliers. The complete sample size is 1000. For calculating the statistics we used reduced sample with size 492, without outliers.

c	c^*	μ	σ	$\frac{\lvert c^*-\mu\rvert}{c^*}$[%]	c^l	c^m	c^u	CI	$\frac{CI}{\mu}$[%]	outliers
c_1	1	2.105	1.347	110.50	0.182	2.082	3.999	3.817	181.31	0
c_2	0.3	2.487	1.191	728.85	0.266	2.637	4.000	3.734	150.16	0
c_3	0.1	0.210	0.025	109.83	0.151	0.213	0.254	0.102	48.73	175
c_4	2.5	3.303	0.409	32.14	2.396	3.327	3.999	1.603	48.53	125
c_5	1	2.058	1.360	105.76	0.107	2.041	3.997	3.890	189.07	0
c_6	0.483	1.552	1.336	221.30	0.191	1.034	3.994	3.803	245.03	0
c_7	0.21	0.914	1.232	335.25	0.006	0.251	3.961	3.954	432.64	0
c_8	3	3.253	0.585	8.42	2.089	3.305	4.000	1.911	58.74	0
c_9	0.1	0.110	0.106	10.00	0.023	0.070	0.430	0.408	370.68	142
c_{10}	0.021	0.527	0.541	2407.80	0.010	0.307	1.852	1.842	349.79	89
c_{11}	1	2.830	0.844	183.05	1.330	2.887	4.000	2.670	94.32	0
c_{12}	3	1.608	0.334	46.39	1.083	1.584	2.400	1.317	81.89	43
c_{13}	0.31	2.859	1.170	822.36	0.540	3.301	4.000	3.460	121.02	0
c_{14}	0.3	0.443	0.168	47.58	0.154	0.442	0.779	0.625	141.23	14
c_{15}	3	2.342	0.755	21.93	1.236	2.170	3.997	2.761	117.89	0
c_{16}	0.483	2.084	1.361	331.56	0.044	2.015	3.999	3.955	189.72	0
c_{17}	0.06	0.629	0.640	948.57	0.000	0.396	2.048	2.048	325.52	9
c_{18}	0.15	0.166	0.095	10.88	0.077	0.132	0.451	0.374	224.91	142
$r_5(0)$	1	0.757	0.671	24.31	0	0.649	1.600	1.600	211.38	0
$R_5(0)$	0.001	0.004	0.005	310.55	0	0.002	0.017	0.017	412.72	72
$r_7(0)$	1	0.640	0.646	36.04	0	0.324	1.600	1.600	250.15	0
$R_7(0)$	0.001	0.001	0.002	43.10	0	5.32E-04	0.007	0.007	517.71	77

Table 8: **Summary of statistics over the estimated parameters by DE on noisy data ($s = 20\%$) regarding the TO observation scenario in a Monte Carlo-based approach**. The column μ represents the mean values of estimated parameters; σ represents the standard deviation of the estimated parameters; $\frac{|c^*-\mu|}{c^*}$ is the relative error; c^l is the 2.5 percentile of the sample values, the lower bound of the 95% confidence interval; c^m is the 50 percentile of the sample values, the median; c^u is the 97.5 percentile of the sample values, the upper bound of the 95% confidence interval; $CI = c^u - c^l$ is the length of the 95% confidence interval; $\frac{CI}{\mu}$ is the confidence interval with respect to the mean; the last column represents the number of outliers. The complete sample size is 1000. For calculating the statistics we used reduced sample with size 564, without outliers.

c	c^*	μ	σ	$\frac{\lvert c^*-\mu\rvert}{c^*}$[%]	c^l	c^m	c^u	CI	$\frac{CI}{\mu}$[%]	outliers
c_1	1	2.388	1.386	138.82	0.230	2.785	3.999	3.770	157.86	0
c_2	0.3	2.781	1.185	827.14	0.312	3.168	4.000	3.688	132.59	0
c_3	0.1	0.165	0.094	65.29	2.06E-05	0.188	0.309	0.309	186.85	129
c_4	2.5	2.924	1.072	16.94	0.162	3.174	4	3.838	131.29	0
c_5	1	2.462	1.423	146.24	0.231	2.856	4.000	3.768	153.04	0
c_6	0.483	2.569	1.408	431.90	0.236	3.158	4.000	3.764	146.50	0
c_7	0.21	0.231	0.273	9.91	0.085	0.137	1.146	1.061	459.63	171
c_8	3	3.305	0.697	10.15	1.796	3.554	4.000	2.204	66.69	0
c_9	0.1	0.111	0.161	11.10	0.011	0.043	0.630	0.620	557.72	126
c_{10}	0.021	0.893	1.128	4154.40	0.017	0.352	3.862	3.845	430.39	44
c_{11}	1	2.796	0.839	179.56	1.127	2.801	4.000	2.872	102.75	0
c_{12}	3	2.399	0.975	20.03	0.779	2.333	3.998	3.219	134.16	0
c_{13}	0.31	2.664	1.169	759.42	0.259	3.023	3.999	3.740	140.38	0
c_{14}	0.3	0.439	0.191	46.36	0.122	0.420	0.882	0.760	173.13	66
c_{15}	3	2.763	0.873	7.92	1.228	2.712	4.000	2.772	100.33	0
c_{16}	0.483	2.532	1.384	424.25	0.236	3.179	3.997	3.761	148.53	0
c_{17}	0.06	0.733	0.614	1122.12	1.36E-04	0.626	1.955	1.955	266.57	0
c_{18}	0.15	0.156	0.160	3.97	0.065	0.103	0.729	0.664	425.79	118
$r_5(0)$	1	0.781	0.370	21.91	5.07E-04	0.856	1.389	1.389	177.85	0
$R_5(0)$	0.001	0.210	0.273	20916.77	0	0.052	0.877	0.877	417.20	9
$r_7(0)$	1	0.982	0.173	1.82	0.626	0.975	1.313	0.686	69.92	70
$R_7(0)$	0.001	0.011	0.024	1010.99	0	0.000	0.092	0.092	829.44	159

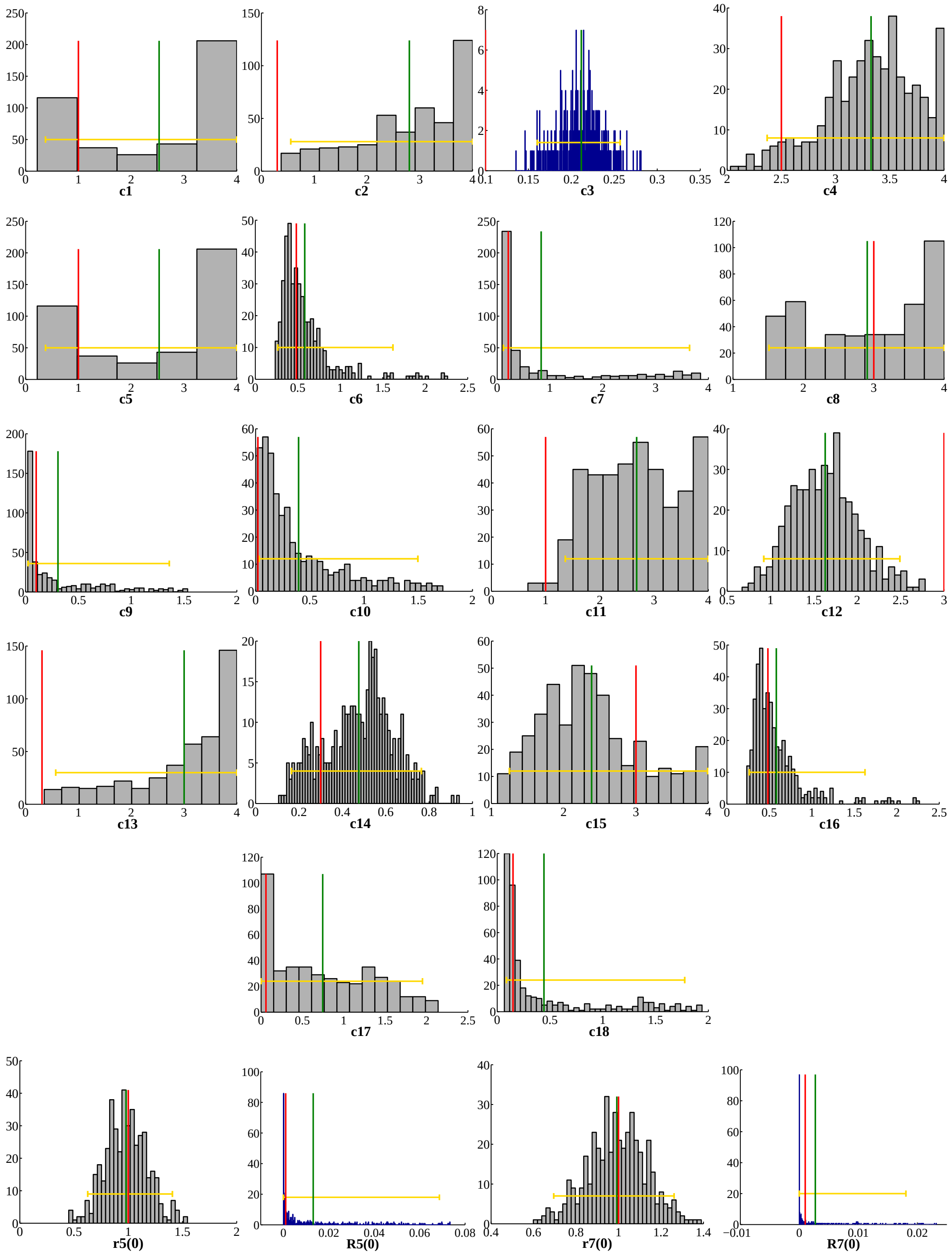

Figure 5: **Histograms of the parameters' estimates by DE on noisy data ($s = 20\%$) regarding the CO observation scenario in a Monte Carlo-based approach.** The red vertical line represents the "true" value of the estimated parameter; the green vertical line represents the mean value of the sample μ; the yellow horizontal line visualizes the uncertainty of the estimated parameter, i.e., its 95% confidence interval. The width of the bins h for a single histogram is calculated according to the Freedman-Diaconis rule $h = \frac{2 \cdot \mathrm{IQR}}{\sqrt[3]{N}}$, where IQR is the interquartile range of the sample and N is the size of the filtered sample (it includes the estimates obtained by those datasets that did not produce any outlier over all parameters), here $N = 428$.

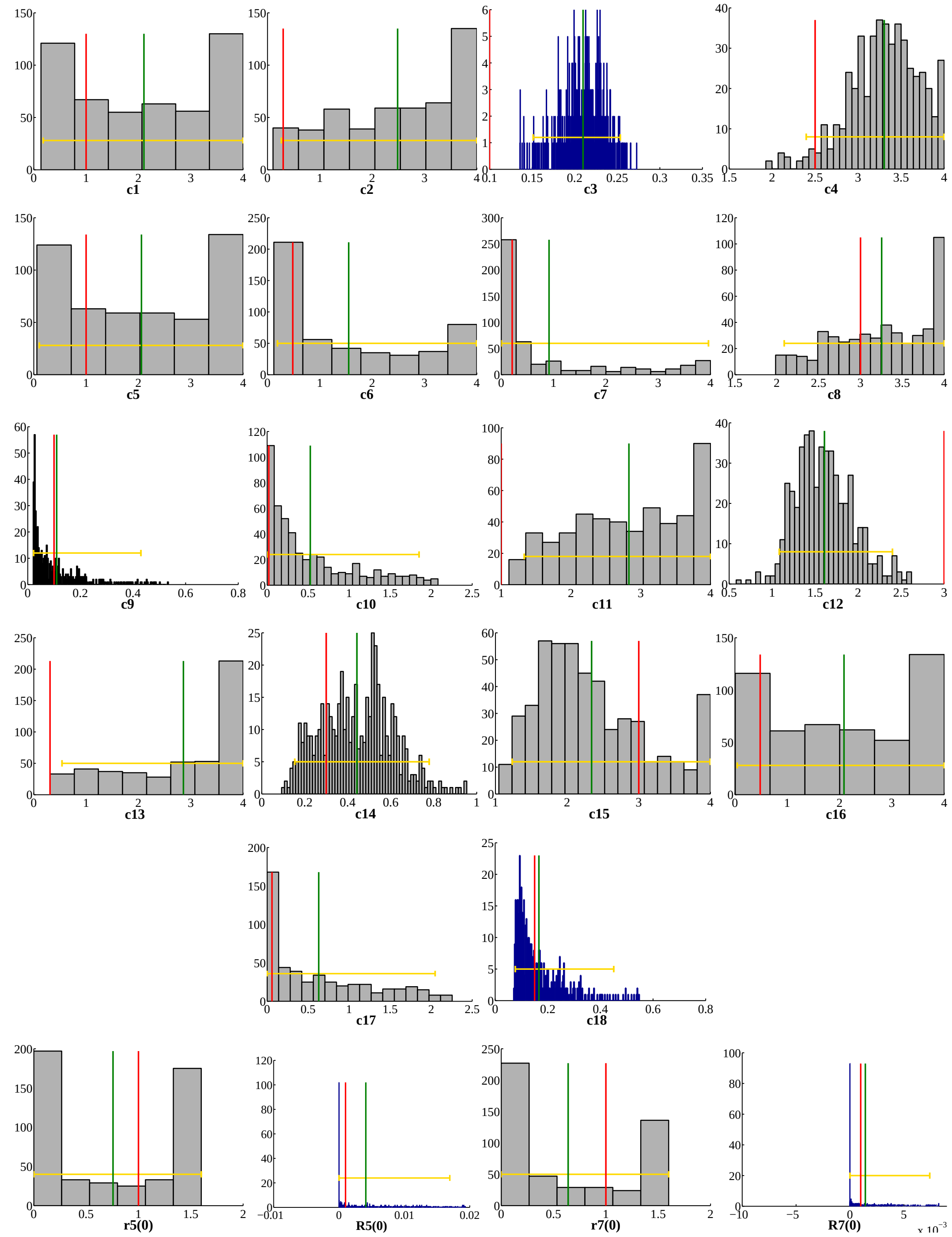

Figure 6: **Histograms of the parameters' estimates by DE on noisy data ($s = 20\%$) regarding the AO observation scenario in a Monte Carlo-based approach.** The red vertical line represents the "true" value of the estimated parameter; the green vertical line represents the mean value of the sample μ; the yellow horizontal line visualizes the uncertainty of the estimated parameter, i.e., its 95% confidence interval. The width of the bins h for a single histogram is calculated according to the Freedman-Diaconis rule $h = \frac{2 \cdot \mathrm{IQR}}{\sqrt[3]{N}}$, where IQR is the interquartile range of the sample and N is the size of the filtered sample (it includes the estimates obtained by those datasets that did not produce any outlier over all parameters), here $N = 492$.

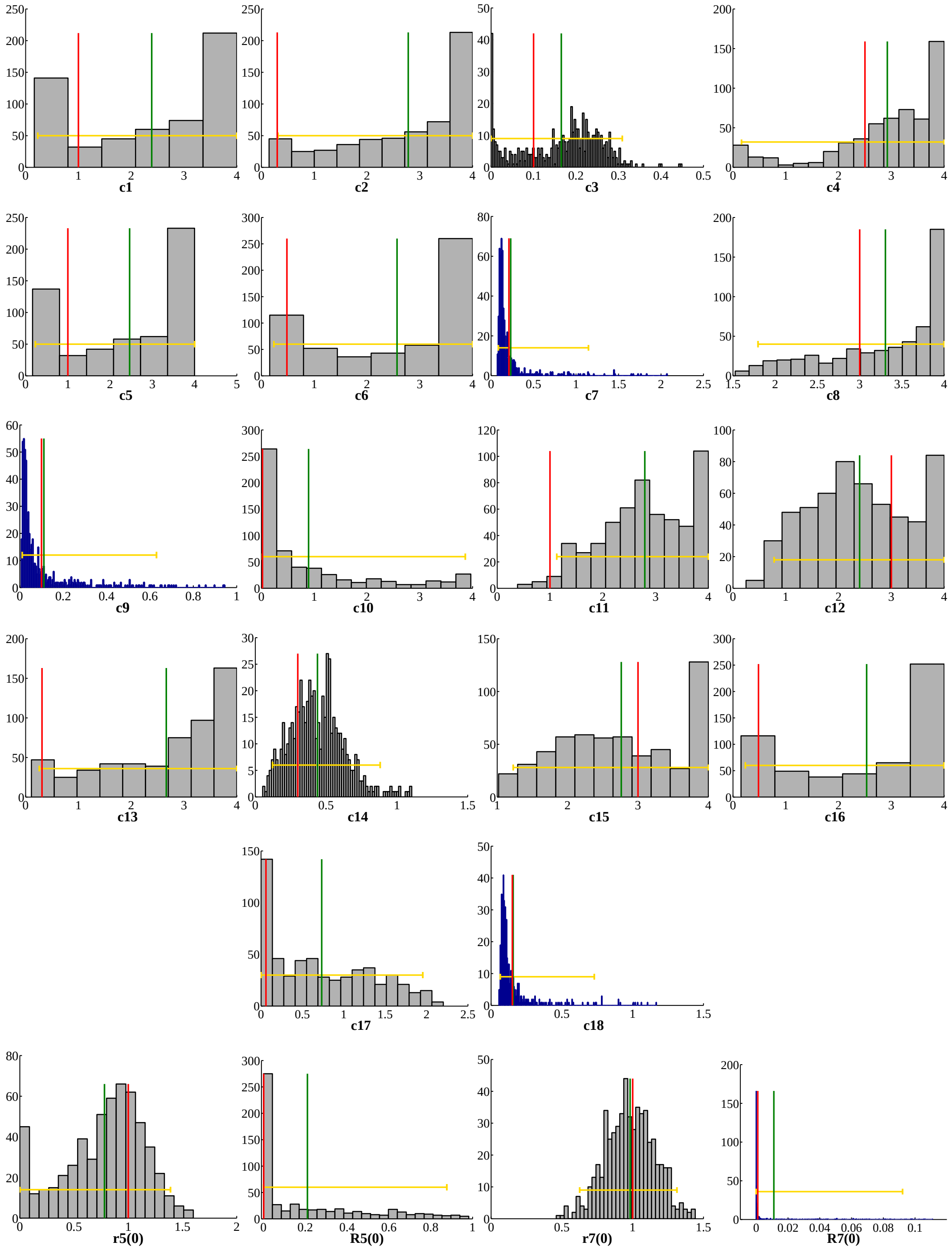

Figure 7: **Histograms of the parameters' estimates by DE on noisy data ($s = 20\%$) regarding the TO observation scenario in a Monte Carlo-based approach.** The red vertical line represents the "true" value of the estimated parameter; the green vertical line represents the mean value of the sample μ; the yellow horizontal line visualizes the uncertainty of the estimated parameter, i.e., 95% confidence interval. The width of the bins h for a single histogram is calculated according to the Freedman-Diaconis rule $h = \frac{2 \cdot \mathrm{IQR}}{\sqrt[3]{N}}$, where IQR is the interquartile range of the sample and N is the size of the filtered sample (it includes the estimates obtained by those datasets that did not produce any outlier over all parameters), here $N = 564$.

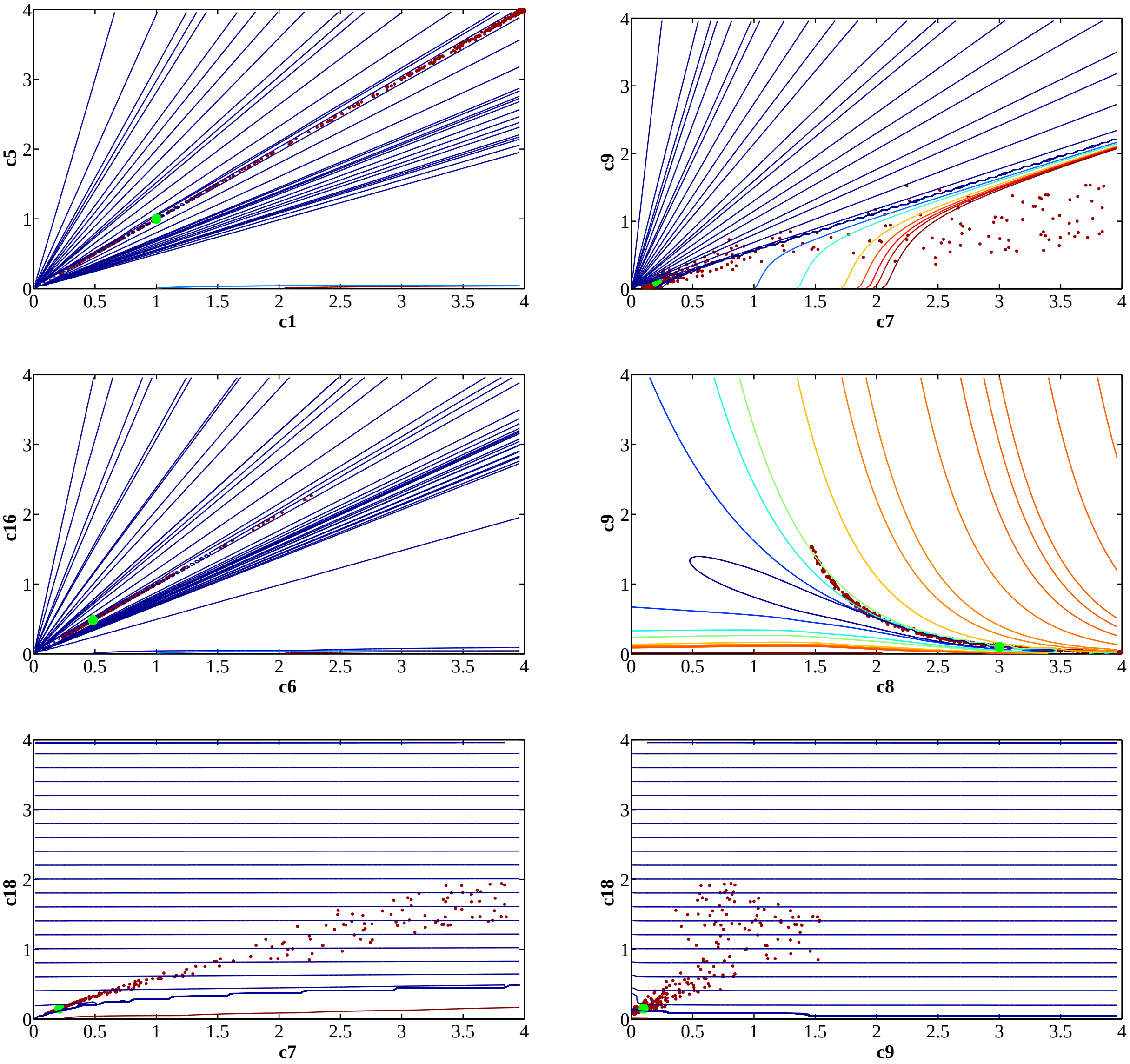

Figure 8: **Contour plots of the objective function with scatter plots of the parameters' estimates obtained by DE on noisy data ($s = 20\%$) regarding the CO observation scenario in a Monte Carlo-based approach.** The plots correspond to the six pairs of the most correlated parameters. The green dot represents the "true" parameter solution (the one used for the simulation generating the artificial data). The red dots are the parameters' estimates obtained by DE with the Monte Carlo-based approach.

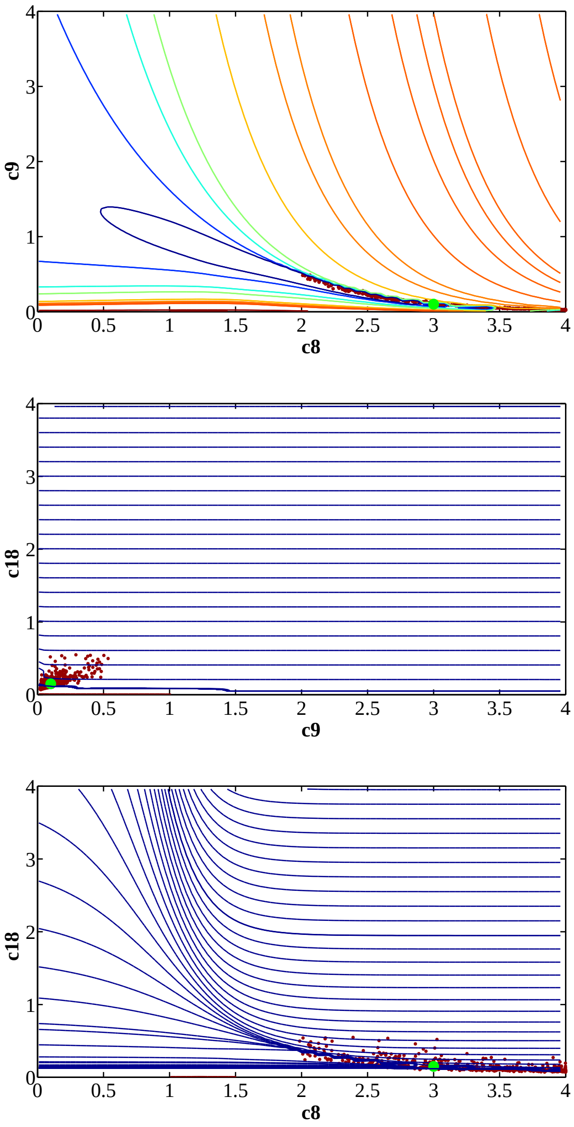

Figure 9: **Contour plots of the objective function with scatter plots of the parameters' estimates obtained by DE on noisy data ($s = 20\%$) regarding the AO observation scenario in a Monte Carlo-based approach.** The plots correspond to the three pairs of the most correlated parameters. The green dot represents the "true" parameter solution (the one used for the simulation generating the artificial data). The red dots are the parameters' estimates obtained by DE with the Monte Carlo-based approach.

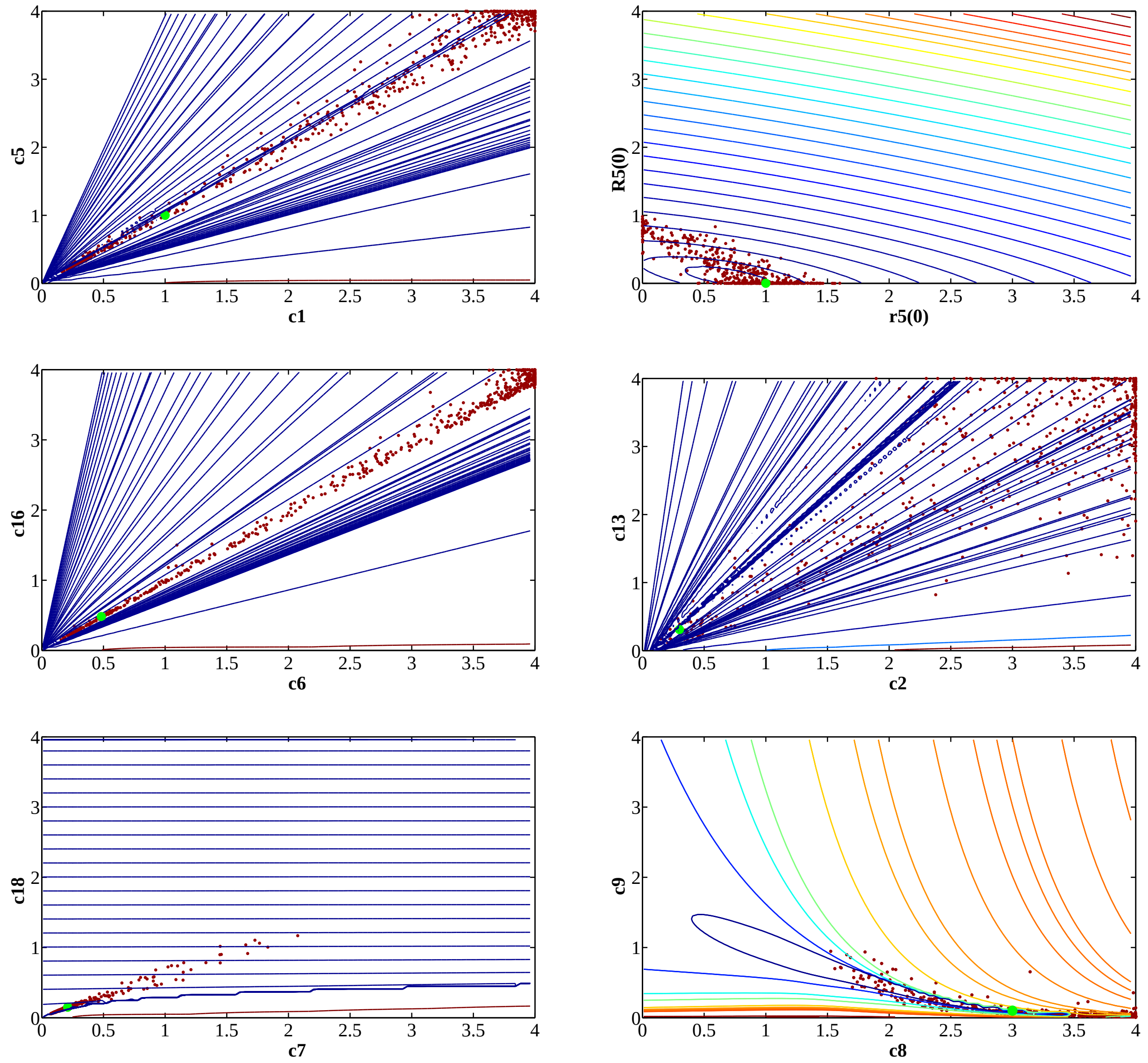

Figure 10: **Contour plots of the objective function with scatter plots of the parameters' estimates obtained by DE on noisy data ($s = 20\%$) regarding the TO observation scenario in a Monte Carlo-based approach.** The plots correspond to the six pairs of the most correlated parameters. The green dot represents the "true" parameter parameters' estimates (the one used for the simulation generating the artificial data). The red dots are the solutions obtained by DE with the Monte Carlo-based approach.
